# Genetic determinants and potential therapeutic targets for pancreatic adenocarcinoma

**DOI:** 10.3389/fphys.2014.00087

**Published:** 2014-03-03

**Authors:** Robert Reznik, Andrew E. Hendifar, Richard Tuli

**Affiliations:** Department of Radiation Oncology, Cedars-Sinai Medical CenterLos Angeles, CA, USA

**Keywords:** pancreatic ductal adenocarcinoma, familiar pancreatic cancer, pancreatic cancer syndromes, pancreatic cancer oncogenes, pancreatic cancer tumor suppressor genes

## Abstract

Pancreatic ductal adenocarcinoma (PDAC) is the fourth leading cause of cancer deaths in both men and women in the United States, carrying a 5-year survival rate of approximately 5%, which is the poorest prognosis of any solid tumor type. Given the dismal prognosis associated with PDAC, a more thorough understanding of risk factors and genetic predisposition has important implications not only for cancer prevention, but also for screening techniques and the development of personalized therapies. While screening of the general population is not recommended or practicable with current diagnostic methods, studies are ongoing to evaluate its usefulness in people with at least 5- to 10-fold increased risk of PDAC. In order to help identify high-risk populations who would be most likely to benefit from early detection screening tests for pancreatic cancer, discovery of additional pancreatic cancer susceptibility genes is crucial. Thus, specific gene-based, gene-product, and marker-based testing for the early detection of pancreatic cancer are currently being developed, with the potential for these to be useful as potential therapeutic targets as well. The goal of this review is to provide an overview of the genetic basis for PDAC with a focus on germline and familial determinants. A discussion of potential therapeutic targets and future directions in screening and treatment is also provided.

## Introduction

Pancreatic ductal adenocarcinoma (PDAC) is the fourth leading cause of cancer deaths in both men and women in the United States, carrying a 5-year survival rate of approximately 5% (Klein, 2012), which is the poorest prognosis of any solid tumor type. Such outcomes are largely due to the fact that 80% of patients have locally advanced or metastatic disease at diagnosis (Siegel et al., 2013). Furthermore, for the 10–20% of patients who present with resectable disease, the overall 5-year survival rate is only 15–20% and median survival is a dismal 18–24 months (Ducreux et al., 2007). PDAC accounts for approximately 90% of pancreatic neoplasms and is synonomous with the term, “pancreatic cancer;” the remaining 15% of pancreatic tumors are represented by acinar cell carcinoma, pancreatoblastoma, solid pseudopapillary neoplasm, serous cystadenoma and pancreatic neuroendocrine tumors (Li et al., 2004a; Hezel et al., 2006; Maitra et al., 2006; Ducreux et al., 2007). Given the dismal prognosis associated with PDAC, a more thorough understanding of risk factors and genetic predisposition has important implications not only for cancer prevention, but also for development of personalized therapies. The goal of this review is to provide an overview of the genetic basis for PDAC with a focus on germline and familial determinants, with a discussion of potential therapeutic targets also provided.

## Inherited risk factors

Increasing knowledge of inherited genetic mutations is leading to a better understanding of pancreatic cancer risk, as these genetic variations are known to contribute to both familial and non-familial (sporadic) PDAC. Studies have estimated up to 10% of patients demonstrate an inherited predisposition to PDAC based on familial clustering (Lynch et al., 1990, 1996; Hruban et al., 1998; Schenk et al., 2001; Del Chiaro et al., 2007; Hruban et al., 2010), while two prospective studies from Sweden and Germany have suggested lower rates of 2.7 and 1.9%, respectively (Hemminki and Li, 2003; Bartsch et al., 2004). A systematic review by Permuth-Wey and Egan revealed the proportion of their study population with a positive family history of pancreatic cancer was only 1.3%. In the latter study, the lower rate was attributed to adjusting for shared environmental factors, such as smoking. Additionally, a majority of the weight (82%) of the meta-analysis was contributed by a prospective cohort study, as opposed to case-control studies, which inherently pose potential for increased biases, such as recall and publication (Permuth-Wey and Egan, 2009).

An inherited predisposition to PDAC is believed to occur in three distinct clinical settings. Firstly, familial cancer syndromes have a well-known association. Peutz-Jeghers Syndrome (PJS), which is associated with germline mutations in the STK11/LKB1 gene, leads to a 36% lifetime risk for pancreatic cancer (Hahn et al., 2003); similarly, Familial Atypical Multiple Mole Melanoma (FAMMM) syndrome, which results due to germline mutations in the p16/CDKN2A gene, leads to an approximate 17% lifetime risk for pancreatic cancer (Hahn et al., 2003); other syndromes include Hereditary Breast-Ovarian Cancer (HBOC) syndrome (BRCA1/2 genes), Hereditary Non-polyposis Colorectal Cancer (HNPCC; mismatch repair genes), and Familial Adenomatous Polyposis (FAP) syndrome (APC gene) (Table 1). Secondly, hereditary causes of pancreatitis, such as the autosomal dominant form caused by germline mutations of the cationic trypsinogen gene, PRSS1, have been indirectly linked to PDAC through early onset chronic pancreatitis with an associated 53-fold increased incidence and approximately 40% of hereditary pancreatitis patients noted to develop pancreatic cancer by age 70 (Hahn et al., 2003; Hezel et al., 2006; Koorstra et al., 2008). Finally, Familial Pancreatic Cancer (FPC) is defined as two or more first-degree relatives having pancreatic cancer without fulfilling criteria for one of the familial cancer syndromes noted above. Although at significantly increased risk for PDAC, pancreatic cancer patients with a hereditary predisposition have not shown any significant difference in clinical course or median survival when compared to sporadic pancreatic cancer patients (James et al., 2004).

**Table 1:** **Pancreatic cancer susceptibility genes**.

**Genes**	**Associated syndrome**	**Freq. of mutation (%)**	**PDAC risk**	**References**
**ONCOGENE**				
BRAF		30		Maitra et al., 2006; Koorstra et al., 2008
AKT2		10–60		Koorstra et al., 2008
KRAS		30–100		Hezel et al., 2006; Koorstra et al., 2008
**TUMOR SUPP**				
BRCA1	HBOC		2.0–2.5×	Thompson et al., 2002; Hahn et al., 2003; Lynch et al., 2008; Ferrone et al., 2009
BRCA2	HBOC	3–10	3.5×	Hahn et al., 2003; Koorstra et al., 2008; Lynch et al., 2008; Klein, 2012
PALB2		3–5	10–32×	Jones et al., 2009; Slater et al., 2010; Schneider et al., 2011; Klein, 2012
PTEN				
p16/CDKN2A	FAMMM	80–95	13–22 × 17% LR	Koorstra et al., 2008; Lynch et al., 2008; Bartsch et al., 2012; Klein, 2012
MMR (MLH1,2)	HNPCC	4	3.7% LR	Koorstra et al., 2008; Bartsch et al., 2012; Klein, 2012
APC	FAP		4.5 × <5% LR	Goggins et al., 2000; Bartsch et al., 2012
TP53	Li-Fraumeni	75–85	7.3×	Koorstra et al., 2008; Bartsch et al., 2012
ATM		<10	2.4×	Maitra et al., 2006; Koorstra et al., 2008
SMAD4/DPC4		50–60		Hezel et al., 2006; Maitra et al., 2006; Koorstra et al., 2008
STK11/KKB1	Peutz-Jeghers		35% lifetime	

*HBOC, hereditary breast and ovarian cancer; FAMMM, familial atypical multiple mole melanoma syndrome; FAP, familial adenomatous polyposis; LR, lifetime risk; HNPCC, hereditary non-polyposis colorectal cancer*.

## Familial pancreatic cancer

The presence of an inherited genetic component and possibility of a hereditary pancreatic cancer syndrome was first suggested by several case reports describing familial aggregation of pancreatic cancers (MacDermott and Kramer, 1973; Reimer et al., 1977; Ehrenthal et al., 1987). Lynch et al performed the first systematic study of 18 families with pancreatic cancer in 1990 and subsequent case-control and cohort studies have shown that individuals with a family history of PDAC are at an increased risk of developing pancreatic cancer themselves (Lynch et al., 1990, 1996; Klein et al., 2001). Furthermore, the odds of having a family history of PDAC are 1.9- to 13-fold higher in pancreatic cancer patients compared to healthy controls (Ghadirian et al., 1991; Jacobs et al., 2010; Klein, 2012). Jacobs et al performed a pooled analysis of data from 5 cohort and one case-control study, which estimated the odds of pancreatic cancer to be 1.76-fold higher (95% CI = 1.19–2.61) among individuals with at least one first-degree relative with PDAC compared to those without a family history. This risk was noted to be even higher in those individuals with at least two first-degree relatives with PDAC with an OR = 4.26 (95% CI = 0.48–37.79) (Jacobs et al., 2010). Tersmette et al similarly noted an increased risk among those with a family history of pancreatic cancer, specifically noting that individuals with a pair of affected first-degree relatives had an 18-fold increased risk of developing PDAC and an estimated lifetime risk of 9–18%, while there was an even more significant 57-fold increased risk in FPC kindred with three or more affected family members when compared to the SEER age-adjusted incidence of pancreatic cancer in the US (Tersmette et al., 2001). The National Familial Pancreas Tumor Registry (NFPTR) has similarly concluded that the risk of pancreatic cancer increases with the number of affected first degree relatives (RR of 6.4 with two first-degree relatives; 32% with three first-degree relatives) (Klein et al., 2004). Based on such conclusive findings, the clinical entity of FPC has been defined (Tersmette et al., 2001; Hahn et al., 2003; Rulyak et al., 2003; Brand et al., 2007; Bartsch et al., 2012; Klein, 2012).

The inheritance pattern of FPC is mostly autosomal dominant and demonstrates a heterogenous phenotype (Slater et al., 2010) The genetic mutations responsible for the majority of clustering in families with PDAC have yet to be identified, although germline mutations in high-penetrance genes such as BRCA2 and PALB2 have been established along with mutations in p16/CDKN2A, STK11/LKB1, PRSS1, BRCA1, mismatch repair genes (hMLH1, hMSH2, hMSH6), VHL, and ATM (Table 1). Despite sharing many of the same genetic mutations associated with well-established familial cancer syndromes, FPC patients must not fulfill criteria for one of the familial cancer syndromes, and thus likely represent phenotypic variants with the associated influence of environmental risk factors. Brand and Lynch noted that the heterogeneity seen within pancreatic cancer cases in both FPC and familial cancer syndromes may indeed be due to the fact these are similar entities that fall along a spectrum. Furthermore, they suggested that the heterogeneity of FPC may actually lie in the varying penetrance of the associated deleterious mutations and the interplay of non-genetic factors, such as environmental risk factors noted above (Brand and Lynch, 2006). The exact relationship between affected family members is also an important indicator of risk and serves as the basis of quantitative risk modeling and prediction tools, such as PancPRO, which is a Bayesian prediction model developed at Johns Hopkins as an extension of BRCAPRO and validated using an FPC registry with an observed-to-predicted pancreatic cancer ratio of 0.83 (Wang et al., 2007; Klein, 2012).

Whereas the incidence of sporadic pancreatic cancer dramatically increases with age, with a peak incidence in the seventh to eighth decade, studies examining the impact of age in FPC patients have yielded inconclusive results. Some studies have suggested a younger age of onset (8–10 years younger) in individuals with a family history or known germline mutation (i.e., BRCA2), while other studies have shown no association with age of onset in those with a hereditary predisposition (Lynch et al., 1990; Phelan et al., 1996; Ozcelik et al., 1997; Hruban et al., 1999; Silverman et al., 1999; Lal et al., 2000; Schenk et al., 2001; Hahn et al., 2003; James et al., 2004; Hezel et al., 2006; Brune et al., 2010; Schneider et al., 2011; Klein, 2012). This may be explained by epigenetic factors and the interaction between genetic and environmental factors in the development of pancreatic cancer (McFaul et al., 2006). This differs from the data on early-onset breast cancer defined as less than 50 years of age, where a conclusively stronger association with predisposing germline mutations, such as BRCA1/2, is seen (Langston et al., 1996; Krainer et al., 1997; Couch et al., 2007). Additionally, BRCA1/2 mutations carriers may be more likely to die from aggressive breast or ovarian cancer at an early age, thereby masking an underlying diagnosis of pancreatic cancer. Interestingly, the phenomenon of “anticipation,” which is a reduction in the age of onset of hereditary pancreatic cancer with successive generations, has been described in 59–85% of FPC families from studies by the European Registry of Hereditary Pancreatitis and Familial Pancreatic Cancer and FaPaCa (German national case collection for FPC) registries (James et al., 2004; McFaul et al., 2006; Schneider et al., 2011). Analysis of 80 affected child-parent pairs by McFaul et al revealed the children died a median of 10 years earlier than the parent, thereby providing strong implications for genetic counseling and secondary screening per the authors (McFaul et al., 2006).

## Pancreatic cancer susceptibility genes

The genetic basis for the majority of PDAC has yet to be discovered. Although several important and high-penetrance genes associated with increased risk of pancreatic cancer have been identified, including BRCA2 and PALB2, it is clear that most cases of pancreatic cancer that demonstrate familial clustering are not explained by known genetic syndromes. This is evident by the fact that only 10–20% of PDAC with familial aggregation results from high-penetrance genes. Novel susceptibility genes in familial aggregating pancreatic cancer still remain to be identified in approximately 80% of affected families, and discovery of such genes is most likely to occur using family-based studies examining linkage or genome-sequencing approaches (Maitra et al., 2006; Bartsch et al., 2012; Klein, 2012).

### Germline mutations

Genes with germline mutations that have been identified in FPC kindred include BRCA2 (and other Fanconi anemia DNA repair pathway genes, including FANCC and FANCG genes), PALB2, PTEN, STK11/LKB1, p16/CDKN2A, TP53, ATM, and PRSS1 (Table 1). Those mutations with high-penetrance, including BRCA2, PALB2, and PTEN, are discussed below. Notably, genetically engineered mouse models have been created for many of these mutations (across a wide range of malignancies), thereby allowing for a tractable *in vivo* system to help determine the biologic impact of oncogenic mutations as well as helping establish genotype-phenotype relationships. Furthermore, these mouse models have the potential to identify early markers of disease and associated genetic mutations, as well as providing improved preclinical models for therapeutic targets and initiatives (Hezel et al., 2006).

### BRCA2 (Fanconi anemia DNA repair pathway gene)

The BRCA2 protein is encoded by the BRCA2 gene located on chromosome 13q and functions in the Fanconi anemia pathway, which is partly responsible for genome-maintenance. Genomic integrity is maintained by enabling homologous recombination (HR)-based double-stranded (DS) DNA repair following inter-strand crosslinking damage, in addition to acting in intra-S phase DNA damage checkpoint control (van der Heijden et al., 2005; Xia et al., 2006). Therefore, BRCA2 mutant cells exhibit defective HR repair, proliferation arrest, impaired cytokinesis, radioresistant DNA synthesis (due to impairment of intra-S phase DNA damage checkpoint control), genomic instability, and hypersensitivity to DNA damaging agents (e.g., PARP inhibitors, mitomycin, platinum, etc.) (Sharan et al., 1997; Patel et al., 1998; Kraakman-van der Zwet et al., 2002; Xia et al., 2006; Couch et al., 2007). The majority (80%) of BRCA2 germline mutations are nonsense or frameshift mutations, such as the 6174delT mutation and other exon 11 mutations, which lead to the development of premature stop codons and result in truncated and non-functional BRCA2 proteins similar to what is seen in BRCA2-mutated breast cancers (Hahn et al., 2003). Additionally, several rare missense mutations have been detected (Couch et al., 2007). Of note, it is estimated that 1% of the Ashkenazi Jewish population in North America harbors the germline BRCA2 6174delT founder mutation, which has been associated with a 10-fold increased risk of developing pancreatic, breast, prostate, and ovarian cancers (Oddoux et al., 1996; Ozcelik et al., 1997) Interestingly though, the BRCA2 6174delT mutation has been described to have independent origins in both Ashkenazi Jewish and non-Jewish populations (Berman et al., 1996; Hahn et al., 2003). BRCA2-deficient murine models of pancreatic cancer have been established in order to evaluate both diagnostic and therapeutic strategies for FPC. In this setting, biallelic loss of BRCA2 alone and certainly in conjunction with p53 deregulation, has been shown to induce the spectrum of pancreatic ductal neoplasia although after a fairly long latency period (Skoulidis et al., 2010; Feldmann et al., 2011; Rowley et al., 2011). The FANCC (located on chromosome 9q) and FANCG (located on chromosome 9p) genes are additional Fanconi complementation group genes which have been implicated in the pathogenesis of pancreatic cancer (Goggins et al., 1996; Maitra et al., 2006).

Previous studies analyzing families with known BRCA2 mutations found young-onset breast and/or ovarian cancer BRCA2 mutation carriers to have a 3.5- to 10-fold increased risk and estimated 5% lifetime risk of developing PDAC relative to non-BRCA2 carriers (Breast Cancer Linkage Consortium, 1999; van Asperen et al., 2005). Although the average age of onset of PDAC does not differ between non-BRCA2 and BRCA2 mutated families, it has been noted that the presence of one young-onset case of PDAC in a pancreatic cancer family may be predictive of the presence of a BRCA2 mutation (Couch et al., 2007) Additionally, it has been suggested that BRCA2 germline mutation carriers exhibit at least two different cancer phenotypes, although it is not yet understood which genetic or environmental factors cause each of these phenotypic variations, it may be due to different mutational loci within the BRCA2 gene (Lubinski et al., 2004; Couch et al., 2007). One phenotype demonstrates a preponderance for breast and ovarian cancers, while a second phenotype is associated with familial pancreatic cancer without an increased incidence of, or high penetrance for, breast and/or ovarian cancer (Couch et al., 2007). The overall prevalence of BRCA2 mutations in moderate-risk (two or more affected first-degree relatives) and high-risk (three or more affected first-degree relatives) pancreatic cancer families, was noted by Couch et al to be approximately 6% with a frequency ranging from 3 to 15% for families depending on the number of affected family members (Couch et al., 2007). Other studies have suggested the prevalence of BRCA2 germline mutations to be significantly higher (12–19%) among individuals with a family history of PDAC, albeit those specifically fulfilling criteria for FPC (Murphy et al., 2002; Hahn et al., 2003). Thus, BRCA2 germline mutations are currently the most frequently identified genetic alteration in FPC even in the absence of breast and/or ovarian cancer (Goggins et al., 1996; Ozcelik et al., 1997; Hahn et al., 2003).

Given that approximately 10% of high-risk FPCs are noted to carry BRCA2-truncating mutations, it has been suggested that these individuals undergo genetic screening for the presence of BRCA2 mutations (Couch et al., 2007). The advantages of clinical testing include the possibility for close monitoring for pancreatic, as well as other BRCA2 mutation-associated cancers (breast, ovarian, prostate) in carriers. Although prophylactic surgical intervention to reduce the risk of breast and ovarian cancer onset is acceptable for BRCA2 mutated female carriers from families with numerous individuals affected with breast and/or ovarian cancers, it is unclear whether there would be risk reduction conferred by similar surgeries in women with BRCA2 mutations from families that only display a history of pancreatic cancer (Couch et al., 2007).

### BRCA1

HBOC is commonly associated with an inherited germline mutation in one of the BRCA1 or BRCA2 alleles with the remaining functional/wildtype allele lost via somatic mutation (Bryant et al., 2005). As previously noted, BRCA2 mutation carriers far outnumber BRCA1 mutation carriers in both HBOC-associated pancreatic cancer and FPC (Hruban et al., 1999; Hahn et al., 2003; Bartsch et al., 2012). The majority of studies examining the prevalence of pancreatic cancer in BRCA1 mutated patients have shown no increased risk, however, others have estimated a 2- to 2.5-fold increased risk (Thompson et al., 2002; Ferrone et al., 2009). Ferrone et al examined 145 Ashkenazi Jewish pancreatic cancer patients and found no increase in frequency of BRCA1 mutations among this group (Ferrone et al., 2009). In addition, an analysis of 66 familial pancreatic cancer patients from NFPTR kindred with three or more relatives with PDAC did not identify any deleterious BRCA1 germline mutations in these patients (Axilbund et al., 2009). In examining whether BRCA1 mutations confer an increased risk of pancreatic cancer, Moran et al studied 268 British BRCA1 mutation-associated HBOC families to determine whether BRCA-mutations conferred an increased risk of PDAC and found no overall increased risk (Moran et al., 2012). In addition, when specifically analyzing for the BRCA1 185delAG founder mutation in pancreatic cancer patients, it was suggested that BRCA1 germline mutations do not contribute to an increased risk of pancreatic cancer (Schnall and Macdonald, 1996).

### PALB2

Germline truncating mutations in the “partner and localizer of BRCA2” (PALB2) gene, which is located on chromosome 16p12 have been identified in approximately 3% of patients with FPC (Jones et al., 2009; Slater et al., 2010). The PALB2 gene encodes for a nuclear protein which co-localizes with BRCA1/2 in nuclear foci, acts as functional bridge between the two proteins, and provides stability to this complex by preventing proteosomal degradation, thereby allowing it to function in HR repair and checkpoint control as part of the Fanconi Anemia DNA repair pathway (Xia et al., 2006). Although it appears that PALB2 allows for stable BRCA2 association with certain nuclear structures, PALB2 is not required for BRCA2 entry into the nucleus. Nearly 50% of nuclear bound BRCA2 is associated with PALB2 and more than 50% of PALB2 is associated with BRCA2, as PALB2 appears to participate in only a subset of cellular responses to DS DNA breaks. Germline BRCA2 missense mutations within the PALB2-binding motif have been shown to disrupt PALB2 binding, thereby disabling BRCA2 function, and PALB2-depleted cells share a phenotype similar to those deficient in BRCA2 function, further highlighting the importance of this complex (Xia et al., 2006). Although mutated PALB2 has been linked with HBOC syndrome and Fanconi Anemia, its role in the pathogenesis of PDAC has only recently been shown. Indeed, according to Slater et al, PALB2 mutation carriers in FPC families demonstrated a 10- to 32-fold increased risk for the development of pancreatic cancer depending on the number of affected family members (Brand et al., 2007; Rahman et al., 2007; Jones et al., 2009; Slater et al., 2010).

Using whole-exome sequencing to examine patients with FPC, Jones et al identified a total of four PALB2 truncating mutations in 3.1% of patients with pancreatic cancer (Jones et al., 2009). While some families with PALB2 stop mutations were noted to have a history of breast and pancreatic cancer, breast cancer was not seen in all families (Jones et al., 2009; Slater et al., 2010). Further studies have identified additional PALB2 mutations in 1–3% of FPC kindred (Tischkowitz et al., 2009; Slater et al., 2010). Nonsense and frameshift mutations, particularly in exon 11 of the PALB2 gene, result in a variety of premature stop codons and ultimately a truncated PALB2 protein, which is exceedingly rare in the general population and those without cancer (Slater et al., 2010). Additionally, while some studies have suggested an earlier age of onset of pancreatic or breast cancer in those with PALB2 mutations in the setting of FPC, recent studies have not observed similar findings (Slater et al., 2010).

### PTEN

Phosphatase and tensin homolog (PTEN) is a major tumor suppressor gene located on chromosome 10q, which encodes a regulator of the NF-kB cytokine network in PDAC. It specifically inhibits activated PI3K (phosphoinositide-3-kinase) and formation of its enzymatic product, phosphorylated phosphatidylinositides (PIP3) (Koorstra et al., 2008). Whereas initiation of PDAC tumorigenesis has been found to be driven by oncogenic KRAS mutations, disease progression has been associated with frequent loss of tumor suppressors within tumor cells, such as the PI3K/PTEN pathway. Possibly due to promoter hypermethylation, aberrant expression and deletion of the PTEN gene has been frequently noted in primary tumor tissue (Asano et al., 2004). Furthermore, the PI3K/PTEN pathway has been reported to be activated in PDAC precursor lesions via activating mutations of PIK3CA, which is the gene that encodes PI3K (Schonleben et al., 2006; Koorstra et al., 2008). Even in the absence of such mutations, it has been observed that the PI3K/AKT pathway is constitutively activated in the majority of pancreatic cancers, through aberrant expression of PTEN as well as amplification or activation of AKT2 kinase (Cheng et al., 1996; Ruggeri et al., 1998; Schlieman et al., 2003; Asano et al., 2004; Reichert et al., 2007; Koorstra et al., 2008). In mouse models, PDAC is driven by combined oncogenic KRAS mutation and haploinsufficient PTEN deficiency, which together promote marked NF-kB activation, its cytokine network (CCL20, CXCL1, IL-6, and IL-23), stromal activation, and immune cell infiltration; these processes shape the pancreatic cancer tumor microenvironment, stimulate the development of peritumoral stroma, and promote local and metastatic progression (Ying et al., 2011). The desmoplastic host response is a hallmark pathologic feature of pancreatic cancer and is characterized by the aforementioned peritumoral stroma consisting of fibroblasts and inflammatory cells. This process is felt to be partly mediated by increased TGF- β levels, and contributes to the decreased tumor vascular density which in turn is felt to compromise delivery of systemic agents and promote radioresistance through hypoxia. As a result, stromal targeting agents are currently under active clinical investigation in patients with locally advanced and metastatic pancreatic cancer (Hezel et al., 2006; Ying et al., 2011). Moreover, constitutively-activated NF-kB and correspondingly upregulated PI3K/AKT signaling have been observed in many primary PDAC cell lines, but not in normal pancreatic tissue specimens suggesting angiogenesis-based pro-survival mechanisms via VEGF, urokinase, and other proinvasive/angiogenic factors (Hezel et al., 2006). Furthermore, activated NF-kB is hypothesized to contribute to pancreatic tumor chemoresistance via upregulation of BCL-2, BCL-XL, and other anti-apoptotic proteins (Hezel et al., 2006).

## Carcinogenesis and somatic mutations

The genetic progression model for PDAC, comparable to that of the adenoma-carcinoma sequence seen in colorectal cancer, results from sequential acquisition of mutations in the proto-oncogene KRAS followed by mutations in tumor suppressor genes such as p16/CDKN2A/INK4A, TP53, and SMAD4 that lead to disturbance in cell cycle regulation, and promote the PanIN-to-PDAC progression (Hruban et al., 2000; Schneider and Schmid, 2003). The noninvasive pancreatic intraepithelial neoplasia (PanIN) lesion may harbor many of the same mutations found in invasive PDAC, although there are likely to be an increasing number of mutations associated with increasing degrees of dysplasia within the PanIN (Hruban et al., 2000). The major genetic alterations leading to sporadic pancreatic cancer are thought to be mutations in the proto-oncogene, KRAS, as well as the p16/CDKN2A/INK4A, TP53, and DPC4/SMAD4 tumor suppressor genes, while mutations in BRCA2, the mismatch repair genes (hMLH1, hMSH2, and hMSH6), and the AKT2 and STK11/LKB1 genes are noted to be rare (Schneider and Schmid, 2003; Hezel et al., 2006). Since p16/CDKN2A and BRCA2 mutations are not detected in the earliest sporadic premalignant pancreatic lesions and are more commonly found in later intermediate and advanced PanIN lesions, supports the hypothesis that these changes likely accumulate and impact the malignant progression of precursor lesions into PDAC rather than participate in cancer initiation (Hezel et al., 2006). It is likely that the relative late event of biallelic loss of BRCA2 in PDAC tumorigenesis is similar and shared between PDAC in those with germline and somatic mutations in the BRCA2 gene (Goggins et al., 2000; Hezel et al., 2006). KRAS gene mutations occur first in the lowest grade of intraductal lesions, known as PanIN-1 and are subsequently followed by p16/CDKN2A gene mutations, which are noted in PanIN-2 (moderately advanced/intermediate grade) lesions; the TP53, SMAD4, and sporadic BRCA2 inactivating mutations are not identified until further progression to a PanIN-3 (high grade) lesion (Hruban et al., 2000; Hezel et al., 2006). Knowledge of the underlying molecular mechanisms involved in pancreatic cancer tumorigenesis will offer new diagnostic and therapeutic options for the treatment and early detection of PDAC and its precursor lesions.

## Oncogenes

Mutated, constitutively activated oncogenes contribute to oncogenesis in PDAC, and include KRAS, BRAF, AKT2, and AIB I (Table 1) (Maitra et al., 2006).

### KRAS

PDAC harbors the highest incidence of mutations in RAS proteins, which are known to mediate pleiotropic effects, including cell proliferation, differentiation, survival, and migration via GTP-binding cytoplasmic protein activity (Schneider and Schmid, 2003; Hezel et al., 2006). Oncogenic KRAS, located on chromosome 12p, is one of the most frequently mutated genes in PDAC with over 90% of tumors harboring a KRAS gene mutation (Hruban et al., 1993; Maitra et al., 2006). The vast majority of the KRAS activating point mutations occur at codon 12 and less frequently at codons 13 and 61, thereby resulting in a constitutively activated protein product and downstream stimulatory signals to RAS effector pathways, such as RAF-mitogen-activated protein (MAP) kinase, PI3K, and RalGDS pathways independent of growth factor stimulation (Hruban et al., 1993; Hezel et al., 2006; Maitra et al., 2006; Koorstra et al., 2008). These mutations appear to occur very early in the development of pancreatic neoplasia, as evidenced by the presence of KRAS mutations in noninvasive precursor lesions, including intraductal papillary mucinous neoplasms (IPMN) and PanINs (Hezel et al., 2006; Maitra et al., 2006). KRAS mutations are the first known genetic alterations known to occur sporadically in normal pancreatic tissue, chronic pancreatitis, and smokers; moreover, they are detected in approximately 30% of early pancreatic neoplasms and close to 100% of advanced PDAC lesions. KRAS-mediated oncogenesis has thus been considered a likely necessary event in the development of PDAC (Rozenblum et al., 1997; Hezel et al., 2006). Biomarker studies have suggested KRAS activation alone is unlikely to single-handedly promote carcinogenesis given the finding of oncogenic KRAS in normal tissues, such as lung, pancreas and colon (Lu et al., 2002). Follow up murine studies have suggested a threshold level of oncogenic KRAS expression is required to initiate transformation through downstream activation of KRAS-effector genes (Ardito et al., 2012; di Magliano and Logsdon, 2013). Although KRAS has been considered an attractive therapeutic target, its specific biochemical properties have made it an elusive target. Oncogenic mutations in KRAS result in a decreased intrinsic rate of GTP hydrolysis and make the molecule insensitive to GTPase activating proteins (GAPs) (Hezel et al., 2006). These oncogenic mutations inhibit the protein's enzymatic activity; thus, an effective KRAS inhibitor would increase the GTPase activity or make the KRAS protein more susceptible to GAPs (Hezel et al., 2006). This differs from the traditional paradigm of attempting to inhibit an oncogene's enzymatic function.

The mammalian Hedgehog family of secreted signaling proteins (Shh, Ihh, and Dhh) regulate the embryonic growth and patterning of many organs, including the pancreas. Activating mutations in Hedgehog proteins have been associated with a variety of cancers. Hedgehog pathway activation, specifically the overexpression of the pathway's principal activating ligand, sonic hedgehog (Shh), has been implicated in both the initiation and maintenance of pancreatic ductal neoplasia as well as more advanced lesions with a relative increase in the expression of Hedgehog ligands observed during pancreatic ductal tumorigenesis. This increased expression of ligands differs from the undetectable expression of Hedgehog ligands in normal human pancreatic ducts. Furthermore, it has been confirmed that the Hedgehog pathway also plays a role in metastases, with inhibition of Hedgehog signaling shown to reduce the incidence of systemic metastasis seen in PDAC xenografts (Berman et al., 2003; Maitra et al., 2006; Koorstra et al., 2008). Studies in pancreatic cancer cell lines have revealed crosstalk between oncogenic KRAS and the Hedgehog signaling pathway, which may suggest oncogenic KRAS plays an important role in activating Hedgehog signaling through the RAF/MEK/MAPK pathway in the absence of Hedgehog ligands during pancreatic tumorigenesis (Koorstra et al., 2008).

### BRAF

The BRAF gene found on chromosome 7q encodes a serine/threonine kinase, which is regulated by binding to RAS and also functions in the RAS-RAF-MEK-ERK-MAP kinase pathway (Koorstra et al., 2008). It is mutated in 1/3 of pancreatic cancers with known wild-type KRAS (Calhoun et al., 2003; Maitra et al., 2006; Koorstra et al., 2008). Thus, KRAS and BRAF oncogenes may function in a mutually exclusive manner in the transformation and carcinogenesis of pancreatic cancers; indeed, some studies suggest that a mutation in one of these two genes invariably results in retention of wild-type copies of the other (Maitra et al., 2006; Koorstra et al., 2008). This suggests a potential requirement for either oncogenic KRAS or BRAF-related signal transduction as a critically important step in the malignant transformation of most pancreatic tumors, and also implies that the RAF-MAP signaling pathway plays a critical role in mediating cancer-causing signals in the RAS pathway (Maitra et al., 2006; Koorstra et al., 2008).

### AKT2

The AKT2 gene is located on chromosome 19q and encodes a serine-threonine kinase that acts as a downstream effector of the PI3K/AKT pathway. This gene is amplified and overexpressed in approximately 10–60% of PDAC (Ruggeri et al., 1998; Schneider and Schmid, 2003; Hezel et al., 2006; Koorstra et al., 2008). It can be activated by epidermal growth factor, platelet-derived growth factor, and basic fibroblast growth factor, all of which are known to be overexpressed in pancreatic cancer, as well as through the PI3K/AKT pathway (Friess et al., 1996; Schneider and Schmid, 2003; Hezel et al., 2006; Koorstra et al., 2008). AKT signaling has also been linked to enhanced insulin-like growth factor I receptor (IGF-IR) expression in PDAC by promoting invasive potential of cells (Tanno et al., 2001; Schneider and Schmid, 2003; Hezel et al., 2006).

### AIB I

Located on chromosome 20q, the AIB I gene is amplified in as many as 60% of PDAC. The nuclear receptor coactivator, Amplified In Breast cancer 1 (AIB I/SRC-3), belongs to the p160/steroid receptor coactivator family (SRC) (Koorstra et al., 2008). AIB I amplification and overexpression are not only detected in hormone-sensitive tumor types, such as breast, ovarian, and prostate cancers, but also in nonsteroid-targeted tumors including colorectal, hepatocellular, and pancreatic cancers (Koorstra et al., 2008).

## Tumor suppressor genes

Tumor suppressor genes are recessive and promote tumor growth when inactivated. Loss of function of several tumor suppressor genes has been observed in PDAC. Biallelic inactivation of these genes can occur via several mechanisms, including intragenic mutation of one allele coupled with loss of the second allele (loss of heterozygosity mutations), deletion of both alleles (homozygous deletion), or hypermethylation of the gene's promotor resulting in silencing of gene expression. The most common tumor suppressor genes noted to be inactivated in greater than half of all PDAC are p16INK4A/CDKN2A, TP53, and SMAD4/DPC4 (Table 1). BRCA2, which is inactivated less frequently (7%), is discussed above (Rozenblum et al., 1997; Hahn and Schmiegel, 1998; Maitra et al., 2006; Koorstra et al., 2008).

### p16INK4A/CDKN2A

The p16INK4A/CDKN2A (cyclin-dependent kinase inhibitor 2A) tumor suppressor gene is located on chromosome 9p and encodes the p16^INK4A^ protein, which regulates the cell cycle through the p16/Rb pathway and controls progression through the G_1_/S transition. Subsequent inhibition of the cyclin D1/CDK4/6 kinase complex results in inappropriate phosphorylation of Rb and blocks entry into S phase of the cell cycle (Schneider and Schmid, 2003; Hezel et al., 2006). Although germline and sporadic mutations have been identified with carriers of the germline p16-Leiden mutation, having an estimated 17% risk of developing pancreatic cancer by the age of 75, CDKN2A has been identified as one of the most frequently inactivated somatic tumor suppressors in PDAC (Koorstra et al., 2008). Inactivation of the gene during sporadic mutation occurs via homozygous deletion in 40% of cases, loss of heterozygosity in 40%, and gene inactivation through promotor hypermethylation in 15–20% (Caldas et al., 1994; Rozenblum et al., 1997; Maitra et al., 2006; Koorstra et al., 2008). p16INK4A/CDKN2A mutations cooperate with KRAS mutations in the development of PDAC, and are known to accelerate tumor progression in the setting of concurrent p53 mutations (Hezel et al., 2006).

Germline mutations in exon 1α of the p16INK4A/CDKN2A gene are associated with FAMMM syndrome (Gruis et al., 1995; Schneider and Schmid, 2003). In addition to a significantly increased risk of developing melanoma, individuals with FAMMM syndrome have a 20- to 34-fold increased risk of developing PDAC, although the penetrance is much lower potentially suggesting a modulating role by environmental factors (Hahn et al., 2003; Schneider and Schmid, 2003; Hezel et al., 2006; Maitra et al., 2006). Homozygous deletions resulting in inactivation of the p16INK4A/CDKN2A gene also frequently inactivate an adjacent gene on chromosome 9p, MTAP (methylthioadenosine phosphorylase), which is located 100 kilobases telomeric and plays an important role in the synthesis of adenosine. As a result of this coincident inactivation, MTAP function is completely lost in approximately 30% of PDAC and is also under active investigation as a potential therapeutic target using purine biosynthesis inhibitors, such as L-alanosine (Hustinx et al., 2005; Maitra et al., 2006; Koorstra et al., 2008). It has been suggested that use of such a targeted agent may be effective against the 1/3 of PDACs that harbor the deletion of this adjacent gene (Hustinx et al., 2005; Maitra et al., 2006; Koorstra et al., 2008).

### TP53

The p53 protein is encoded by the TP53 gene located on chromosome 17p and is responsible for modulating cellular responses to cytotoxic stress by maintaining genomic stability. Specifically, p53 is responsible for regulation of the G_1_/S cell cycle checkpoint, maintenance of G_2_/M arrest, induction of apoptosis, and protection against genomic rearrangement and accumulation of mutations. It also suppresses cellular transformation caused by oncogenic activation or loss of tumor suppressor pathways; thus, deletion or inactivation of TP53 is associated with aneuploidy, as well as the growth and survival of cells harboring chromosomal aberrations and genetic instability with potential for carcinogenic transformation (Schneider and Schmid, 2003; Hezel et al., 2006; Maitra et al., 2006; Koorstra et al., 2008). The loss of p53 function results in deregulation of two essential controls over cell number, cell proliferation, cell division and cell death (Schneider and Schmid, 2003; Hezel et al., 2006). Notably, TP53 inactivation is the most common somatic alteration in human cancer, has been described in Li-Fraumeni syndrome, and is inactivated in 75–85% of PDAC almost always via intragenic mutation coupled with a somatically acquired loss of the second allele (Redston et al., 1994; Rozenblum et al., 1997; Schneider and Schmid, 2003; Hezel et al., 2006).

Additionally, p53-induced growth arrest is achieved by transactivation of p21. Binding of p53 to DNA stimulates p21 protein production, which negatively regulates the cyclin D/CDK2 complex and prevents the cell from progressing through G_1_-S phase. This process also allows time for damaged DNA to be repaired. However, mutated p53 is unable to bind DNA, p21 is not available, and abnormal and deregulated growth occurs as a result. Loss of p21 activity through lack of transactivation has been observed in approximately 30–60% of PDAC specimens (Koorstra et al., 2008).

### SMAD4

The SMAD4 gene, also known as DPC4 (deleted in pancreatic carcinoma, locus 4) is located on chromosome 18q21 and is inactivated in approximately 50–60% of PDAC (Hezel et al., 2006; Maitra et al., 2006; Jones et al., 2008). In 30–35% of the tumors, the gene is inactivated by homozygous deletion and by a loss of heterozygosity mutation in another 20–30% of cases. The protein product of the SMAD4 gene functions in transcriptional regulation and localizes to the nucleus following activation of the TGF-β intracellular signaling cascade (Derynck and Zhang, 2003; Hezel et al., 2006; Maitra et al., 2006). Once Smad4 is in the nucleus, it exhibits growth-controlling effects by regulating expression of specific gene targets (Maitra et al., 2006). Loss of SMAD4 interferes with the intracellular signaling cascades downstream from TGF-β, resulting in decreased growth inhibition via loss of pro-apoptotic signaling or via inappropriate G_1_/S transition (Koorstra et al., 2008). Although it is assumed that the growth-inhibitory function of TGF-β is important in SMAD4 tumor suppressor activity, data has also suggested a TGF-β independent function of SMAD4, which modulates the interaction of the tumor with the microenvironment. This includes a decrease in pro-angiogenic VEGF expression and an increase in angiogenesis inhibitor TSP-1 (Schneider and Schmid, 2003). Thus, SMAD4 tumor suppressor function may also occur through regulation of an angiogenic mechanism (Schneider and Schmid, 2003; Hezel et al., 2006). Frequent inactivation of the SMAD4 gene appears to be specific to PDAC, as inactivation is rarely noted in other tumor types or in non-ductal neoplasms of the pancreas (Maitra et al., 2006; Koorstra et al., 2008). Immunohistochemical staining for the Smad4 protein on tissue sections correlates strongly with SMAD4 gene status; thus, immunostaining can be used diagnostically to determine SMAD4 gene status in biopsies and resected tissues, as well as to suggest a pancreatic primary in the setting of occult metastatic adenocarcinoma (Wilentz et al., 2000; Maitra et al., 2006; Koorstra et al., 2008). This is particularly useful since PDAC with loss of Smad4 reportedly demonstrate an increased propensity for distant metastases and thus have a generally poorer prognosis, although SMAD4 gene status is not yet utilized for prognostic stratification (Schneider and Schmid, 2003; Blackford et al., 2009).

### STK11/LKB1 and other tumor suppressor genes

Genetic alterations in tumor suppressor genes found in lower frequency (<10%) in pancreatic cancer include STK11/LKB1, MKK4, TGFβ R1 (ALK 5, chromosome 9q), TGFβ R2 (chromosome 3p), ACVR1β (ALK 4, chromosome 12q), ACVR2 (chromosome 2q), FBXW7 (CDC4), EP300, BRCA2, ATM, and AKT2 (Maitra et al., 2006; Koorstra et al., 2008). These infrequently mutated genes provide further insight into cellular pathways altered in PDAC, as well as potential therapeutic targets for gene-specific therapies (Maitra et al., 2006). The STK11/LKB1 gene on chromosome 19p13 encodes for a serine/threonine kinase that regulates cell polarity and metabolism (Jenne et al., 1998; Schneider and Schmid, 2003; Hezel et al., 2006). Inactivation of the STK11 gene appears to play a role in both hereditary and sporadic PDAC (Schneider and Schmid, 2003; Hezel et al., 2006). Germline mutations in this gene are associated with PJS and are identified in approximately 50% of PJS families who typically present with hamartomatous polyps of the GI tract, pigmented macules of the lips and buccal mucosa, as well as a 36% lifetime risk for the development of pancreatic cancer (>40-fold increased RR) (Giardiello et al., 2000; Sato et al., 2001; Hahn et al., 2003; Hezel et al., 2006). Somatic STK11 mutations have been observed in approximately 5% of sporadic PDAC, particularly those that arise in association within an IPMN, whereas loss of heterozygosity is seen in approximately 25% of patients with IPMN who lack PJS features (Sato et al., 2001; Hezel et al., 2006).

In a smaller percentage of PDAC, intragenic mutations and homozygous deletions of the MKK4 gene are noted. This gene encodes for a component of a stress-activated protein kinase cascade and functions in apoptosis and growth control (Koorstra et al., 2008). MKK4 is preferentially inactivated in specific subsets of pancreatic cancer metastases and less commonly in the primary tumors of the same patients (Xin et al., 2004; Koorstra et al., 2008). Thus, it has been suggested that the MKK4 protein product may function as a suppressor of metastasis in pancreatic cancer, as it is does in breast and prostatic carcinomas (Xin et al., 2004). There is also a noted trend toward worse survival in those patients with loss of MKK4 expression and thus evaluation of MKK4 immunolabeling may have prognostic value. Furthermore, there lies the potential for MKK4 to be a therapeutic target for restoration of the stress-activated protein kinase pathway in advanced PDAC patients (Xin et al., 2004; Koorstra et al., 2008).

## Biomarkers and therapeutic targets

A better understanding of the genetic causes of sporadic and FPC has afforded the opportunity to investigate novel mechanism-based targeted and systemic therapies, as well as predictive and prognostic biomarkers.

### Targeting DNA repair

BRCA1/2, other Fanconi anemia family proteins, and PARP-1/2 among others, function in a coordinated series of early events in DNA damage repair. When this process is impaired, cells become exquisitely sensitive to DNA damaging agents. The potential to exploit this strategy exists in PDAC. Mitomycin C is an alkylating antineoplastic agent that works by inducing interstrand DNA crosslinking with eventual production of DS-breaks. Early xenograft studies by van der Heijden et al showed a more pronounced response of FANCC and BRCA2-deficient pancreatic tumors to mitomycin C relative to Fanconi anemia proficient xenografts (van der Heijden et al., 2005). This enhanced preclinical response to mitomycin C involved cell cycle arrest in late S or G_2_/M phase and caspase-dependent apoptosis; similar findings were noted with cyclophosphamide, another DNA interstrand crosslinking agent (van der Heijden et al., 2005). In spite of such promising preclinical data, clinical results with both mitomycin C and cyclophosphamide have been disappointing. In a retrospective analysis by Brunner et al, the combination of 5-fluorouracil and mitomycin C-based chemoradiotherapy demonstrated worse median overall survival (9.7 vs. 12.7 months) and 1 year overall survival (53 vs. 40%) compared to gemcitabine and cisplatin-based chemoradiotherapy in patients with locally advanced PDAC with similar toxicities. (Brunner et al., 2011). A phase II study by Cereda et al looking at salvage therapy with mitomycin C and ifosfamide (analog of cyclophosphamide) in gemcitabine-resistant metastatic pancreatic cancer was closed prematurely based on poor clinical outcomes with 71% of patients experiencing chemotherapy interruption due to progressive disease and 80% of patients demonstrating grade >2 toxicity. This study concluded that the mitomycin C and ifosfamide regimen was considered insufficiently active in gemcitabine-resistant metastatic pancreatic cancer (Cereda et al., 2011).

Poly (ADP-ribose) polymerase-1/2 (PARP-1/2) activity and poly (ADP-ribose) polymerization are essential for the repair of single stranded (SS)-DNA breaks through the base excision repair (BER) pathways (Bryant et al., 2005). Additionally, enhanced PARP-1 expression is seen in many tumor types compared to normal cells, and represents one of the mechanisms by which tumors avoid apoptosis caused by DNA damaging agents (Berger et al., 1978). In the absence of PARP-1, spontaneous SS-breaks can collapse replication forks and trigger HR repair (Bryant et al., 2005). Despite its role in cellular responses to genotoxic stress, in knockout mouse models, PARP-1 has been shown to not be required for survival or fertility in the absence of such insults; thus, PARP-1 can be considered a non-essential DNA repair protein in the setting of functional HR repair mechanisms (Bryant et al., 2005). Inhibition of PARP is therefore known to sensitize tumor cells to cytotoxic agents, such as topoisomerase-I inhibitors and alkylators, which induce DNA damage normally repaired by BER. The resulting increase in HR repair that occurs in PARP-1 deficient mice is felt to represent an error-free mechanism, which likely explains why the genetic instability in PARP-1 deficient cells is not associated with accumulation of mutations or cancers (Bryant et al., 2005). BRCA2 and other Fanconi Anemia-pathway defective cells are felt to be sensitive to single-agent PARP inhibition and restoring functional BRCA abrogates this activity. (Bryant et al., 2005). Therefore, compromise of both BER and HR repair is felt to result in a lethal persistence and accumulation of recombinogenic lesions, chromosomal instability, cell cycle arrest, and resulting apoptosis partly through use of alternative error-prone repair pathways, such as SS annealing and non-homologous end joining (NHEJ) (Farmer et al., 2005). Bryant et al demonstrated that PARP inhibitors were profoundly cytotoxic to a BRCA2-deficient cell line at low concentrations relative to BRCA2-competent cells and normal cells, thus suggesting potential for a wide therapeutic index (Bryant et al., 2005). Whereas clonogenic survival was significantly reduced following PARP-1 and BRCA2 protein co-depletion in human cells using siRNA, depletion of PARP-2 with BRCA2 had no effect on clonogenic survival following treatment with PARP inhibitors. Depletion of PARP-2 in PARP-1- and BRCA2-depleted cells also did not result in added toxicity. Thus, it has been suggested that PARP-1, rather than PARP-2, is responsible for protection against spontaneously occurring recombinogenic lesions in cells. In turn, these lesions may convert to persistent DS breaks and collapsed replication forks during replication, which may ultimately result in cellular apoptosis in the absence of PARP-1-mediated repair (Bryant et al., 2005). Preclinical studies have demonstrated the potential effectiveness of PARP inhibitors in targeting pancreatic cancers demonstrating biallelic inactivation of the ATM gene and clinical trials are underway investigating the role of PARP-inhibition with DNA damaging agents in patients with or without BRCA-mutations (NCT01908478, NCT01585805) (Williamson et al., 2012).

### Hedgehog signaling pathway inhibition

Aberrant activation of previously quiescent developmental signaling pathways, such as the Hedgehog pathway has been implicated in PDAC tumorigenesis, progression and development of metastases (Koorstra et al., 2008). Targeting of sonic hedgehog, the overexpressed principal activating ligand of the Hedgehog signaling pathway, has been a focus of much investigation (Berman et al., 2003; Maitra et al., 2006). Drugs such as cyclopamine have been developed which specifically inhibit the hedgehog pathway, thereby producing dramatic anti-tumor effects in murine xenograft PDAC models without significant side effects (Berman et al., 2003; Maitra et al., 2006). Given the dramatic results seen with cyclopamine, development of additional inhibitors of the hedgehog pathway, such as IPI-926 and GDC-0449, have and continue to be explored in clinical studies in the treatment of both pancreatic and other solid tumors with varied responses noted thus far (Berman et al., 2003; Maitra et al., 2006; Kelleher, 2011; LoRusso et al., 2011). One such study by Olive et al, examined the addition of IPI-926 to gemcitabine applied in a genetically engineered pancreatic cancermouse model, which demonstrated a significant depletion of tumor-associated stroma and a corresponding increased intratumoral concentration of gemcitabine (Olive et al., 2009). Unfortunately, a follow-up double blind, placebo-controlled phase II study randomizing patients with previously untreated metastatic pancreatic cancer to gemcitabine with or without IPI-926 (saridegib) was discontinued following interim analysis due to inferior survival of the investigational arm. As a result, clinical excitement over hedgehog inhibition has waned. Additionally, given the relationship between the RAS/MAPK and Hedgehog signaling pathways in PDAC, it has been suggested that synergistic targeting of both the RAS and Hedgehog pathways may represent a new therapeutic strategy for the treatment of PDAC (Pasca di Magliano et al., 2006; Koorstra et al., 2008; Mimeault and Batra, 2010; LoRusso et al., 2011).

### KRAS and beyond

Benzodiazepine peptidomimetics have been shown to block the post-translational attachment of farnesyl groups to Ras proteins, which are required for attachment to the cellular membrane. In preclinical studies, such farnyltransferase inhibitors restored normal growth patterns to Ras-transformed cells suggesting therapeutic potential in PDAC (James et al., 1993). However, when this was examined in a Phase III clinical study, the addition of tipifarnib (farnyltransferase inhibitor) to gemcitabine in advanced pancreatic cancer did not demonstrate any improvement in overall, 6-month, and 1-year survivals over gemcitabine alone, with acceptable toxicity noted in both arms (Van Cutsem et al., 2004). Based on the significant clinical benefit noted in patients with locally advanced disease, it was suggested that the negative results of this study could be explained by 76% of the patients in the study having metastatic disease and correspondingly large tumor burdens (Van Cutsem et al., 2004). As noted previously, tumors with oncogenic KRAS are often associated with relative drug resistance and poor prognosis (Hezel et al., 2006; Barbie et al., 2009). Thus, the combination of oncogenic KRAS mutation with PTEN-deficiency seen in PDAC promote NF-kB activation and sustained activity of the NF-kB downstream cytokine pathway. This is mediated via an elevated PI3K pathway, which provides yet an additional avenue for targeted therapies for those tumors demonstrating altered PI3K regulation (Ying et al., 2011).

Targeting of KRAS effectors such as mTOR, which act downstream of AKT2 have previously been shown to be activated in PDAC. Targeting of mTOR with an mTOR inhibitor (rapamycin analog) has shown tumor growth inhibition in several PDAC cell lines (Asano et al., 2005; Hezel et al., 2006). Additionally, rapamycin has been shown to inhibit PDAC xenograft growth and metastasis (Bruns et al., 2004). The possible mechanisms by which these agents work include induction of endothelial cell death and tumor vessel thrombosis (Bruns et al., 2004). A phase II study by Wolpin et al examined the use of everolimus in gemcitabine-refractory, metastatic PDAC patients. Single-agent everolimus was well-tolerated, but showed minimal clinical activity with no clear evidence of treatment response, only 21% of patients having stable disease at 2 months, a median PFS of 1.8 months, and an overall survival of 4.5 months (Wolpin et al., 2009).

Dramatic tumor shrinkage was noted in a recent mutated KRAS lung cancer model when treated with a combination of a dual PI3K/mTOR inhibitor and a MEK (MAP/ERK kinase) inhibitor (Engelman et al., 2008). This provides preclinical feasibility of the concept that targeting KRAS surrogates and downstream targets is potentially a feasible therapeutic strategy. As a result, numerous IV and oral PI3K and MEK inhibitors are in various stages of clinical development and testing (Phase I and II) (Engelman et al., 2008; Ying et al., 2011; Britten, 2013). Given the known cooperation between oncogenic KRAS and PTEN deficiency in PDAC tumorigenesis, further investigation is validated for combined therapies with MEK inhibitors and PI3K or NF-kB inhibitors. This concept of combination therapies with multiple targets is further supported by the poor results seen in Phase I/II studies of single-agent MEK inhibitors (CI-1040, selumetinib), which have shown minimal clinical response and only a marginal improvement in median survival when used alone (Rinehart et al., 2004; Lorusso et al., 2005; Bodoky et al., 2012; Britten, 2013).

### Growth factor inhibition

The epidermal growth factor receptor (EGFR), which consists of 4 separate receptors [HER1 (ErbB-1), HER-2/Neu (ErbB-2), HER-3 (ErbB-3), and HER-4 (ErbB-4)], is overexpressed and plays a distinct role in PDAC (Koorstra et al., 2008). HER-2/neu overexpression is most prominent in well-differentiated PDAC and early-stage precursor lesions, and appears to correlate with degree of dysplasia in the latter (Koorstra et al., 2008). In PDAC, HER-2/neu amplification has been observed in 10–60% of patients. (Stoecklein et al., 2004; Talar-Wojnarowska and Malecka-Panas, 2006; Koorstra et al., 2008). This gene amplication is of interest as it could potentially be a target of trastuzumab, the monoclonal antibody directed against the HER2/neu receptor. (Koorstra et al., 2008). In addition, increased levels of fibroblast growth factor (FGF), FGF-receptor, insulin-like growth factor I (IGF-I), IGF-I receptor, nerve growth factor, and vascular endothelial growth factor (VEGF) have also been reported in PDAC. Targeted therapies directed toward several of these growth factors have been examined with some under active clinical investigation, as noted below (Koorstra et al., 2008).

### EGFR

Despite the complexity of the EGFR signaling cascade, which is known to provide a multitude of resistance mechanisms to EGFR-targeted agents in PDAC, the small molecule tyrosine kinase inhibitor (TKI) of EGFR, erlotinib, has been approved in the US. Moore et al conducted a phase III double-blind study randomizing patients with advanced PDAC to gemcitabine with or without erlotinib. A small but statistically significant improvement in PFS, one-year OS, and median OS was seen (Bruns et al., 2000; Li et al., 2004b; Ducreux et al., 2007; Moore et al., 2007). Interestingly, the subset of patients who developed erlotinib-related skin toxicity had a significantly more profound clinical response. It has been hypothesized that these results may be due to a decrease in tumor vasculature mediated through endothelial apoptosis, given that EGFR is expressed not only on tumor cells but also on dividing endothelial cells (Bruns et al., 2000; Li et al., 2004b; Ducreux et al., 2007). Furthermore, the effect of erlotinib may also potentially be due to inhibition of proangiogenic factors (VEGF, IL-8) by EGFR inhibitors, given that activation of the EGF receptor on tumor cells is known to induce the production of VEGF (Bruns et al., 2000; Li et al., 2004b; Ducreux et al., 2007). Attempts to correlate expression of molecular targets, such as EGFR, to outcomes in erlotinib-based therapies have been unsuccessful to date (da Cunha Santos et al., 2010). EGFR expression as quantified by immunohistochemistry techniques is unlikely to identify those tumors predominantly driven by the EGFR signaling pathway and thus would potentially be responsive to EGFR inhibition (Philip et al., 2010). A phase III study investigated the addition of cetuximab to gemcitabine in an unselected patient population (not selected for presence of EGFR mutations) and found no significant improvement in overall or progression-free survival observed relative to gemcitabine alone (Ducreux et al., 2007; Philip et al., 2010). Ongoing and future research focusing on identification of molecular predictors of resistance and sensitivity to EGFR blockade will potentially improve our understanding of such therapies and selected patient response (Philip et al., 2010).

### SMAD4/DPC4

Iacobuzio-Donahue et al recently reported on Smad4 as a potential predictor of local vs. distant failure using rapid autopsy specimen of patients with PDAC (Iacobuzio-Donahue et al., 2009). Interestingly, intact Smad4 immunolabeling strongly correlated with a locally destructive phenotype (*p* = 0.007) and cause of death was attributed to local progression in 30% of patients. In a prospective single arm study of locally advanced PDAC patients treated with induction cetuximab, gemcitabine, and oxaliplatin followed by cetuximab, capecitabine, and radiotherapy, Crane et al similarly found Smad4 expression correlated with local rather than distant disease progression and potentially represented a predictive biomarker (Crane et al., 2011). Based on these results, RTOG 1201 is currently randomizing patients to upfront gemcitabine followed by high-intensity capecitabine-based IMRT (63.0 Gy) vs. upfront gemcitabine followed by standard intensity capecitabine-based 3D-CRT (50.4 Gy) vs. upfront FOLFIRINOX followed by standard intensity capecitabine-based 3D-CRT (50.4 Gy) and stratifying patients for intensification of local therapy based on Smad4 status (NCT01921751).

### VEGF

It is well-known that VEGF and VEGFR are frequently overexpressed in PDAC. Disruption of VEGF signaling and tumor angiogenesis using soluble VEGFR, VEGF high-affinity binding chimeras, anti-VEGF monoclonal antibodies (e.g., bevacizumab), and ribozymes have shown strong antitumor activity in PDAC mouse xenografts and cultured pancreatic cancer cell lines (Hezel et al., 2006; Koorstra et al., 2008). Unfortunately, clinical results have been disparate. Kindler et al conducted a single arm phase II study investigating the addition of bevacizumab to gemcitabine in patients with advanced PDAC and noted a promising median OS of 8.8 months, PR of 21%, and *SD* of 46% (Kindler et al., 2005). The follow up CALGB phase III placebo-controlled study randomized patients to gemcitabine with or without bevacizumab and found no statistically significant improvement in clinical outcomes (Kindler et al., 2010). Similar disappointing results have been noted with small molecule TKI of VEGFR1-3, axitinib. (Spano et al., 2008). In addition, based on the enhanced radiosensitization seen with the addition of bevacizumab to 5-FU-based radiotherapy in rectal cancer, similar strategies for radiosensitization could be considered in the treatment of PDAC (Willett et al., 2006; Ducreux et al., 2007). A phase I study evaluating the safety of bevacizumab with concurrent capecitabine-based radiotherapy in locally advanced PDAC initially showed bevacizumab-related GI toxicity of duodenal mucosal ulceration, bleeding, and perforation, with protocol mandated dose reductions in capecitabine required in 43% of patients, thus concluding that further study of bevacizumab with chemoradiotherapy was indicated (Crane et al., 2006). A future therapeutic target may be the VEGF-C, a regulator of lymphangiogenesis, which is noted to be overexpressed in PDAC, and may contribute to the lymphatic spread and metastasis that are commonly seen in pancreatic cancer (Hezel et al., 2006).

### IGF-I

Elevated expression of IGF-I has been noted in PDAC tumor cells and their surrounding stroma (Hezel et al., 2006). In addition, there is aberrant activation and constitutive overexpression of the IGF-I receptor (IGF-IR) in approximately 64% of pancreatic tumor cells (Bergmann et al., 1995; Hakam et al., 2003; Ouban et al., 2003; Stoeltzing et al., 2003; Hezel et al., 2006). Furthermore, in a particular PDAC cell line, aberrant expression and activation of IGF-IR via paracrine and autocrine IGF-I signaling was noted to promote cell proliferation and growth-factor-independent survival (Nair et al., 2001). Therefore, inhibition of this pathway using anti-IGF-IR antibodies or expression of truncated IGF-I receptors (via recombinant adenovirus technique) that function as a dominant-negative form of IGF-IR has been examined in the preclinical setting and shown to inhibit the growth of xenograft tumors by up-regulating stressor induced apoptosis, blocking IGF-I and IGF-II induced activation of AKT-1, as well as sensitizing tumor cells to chemotherapy (Maloney et al., 2003; Min et al., 2003; Hezel et al., 2006). Given the encouraging preclinical results, several phase I and II studies of IGF-IR monoclonal antibody and small molecule agents have been pursued (Carboni et al., 2009; Hewish et al., 2009; Kindler et al., 2012). Kindler et al performed a randomized phase II study of ganitumab (AMG 479; monoclonal antibody antagonist of IGF-IR) and gemcitabine vs. gemcitabine and placebo in previously untreated metastatic PDAC. The ganitumab arm demonstrated acceptable toxicity, as well as trends toward improved 6- and 12-month survival, PFS, and overall survival. Given these favorable results, a randomized Phase III study of AMG 479 and gemcitabine in metastatic pancreatic adenocarcinoma was pursued, randomizing patients to AMG 479 (12 or 20 mg/kg) and gemcitabine vs. placebo and gemcitabine. Unfortunately, this study was stopped early for futility based on pre-planned interim analysis (NCT01231347).

## Future directions/screening

While screening of the general population is not practicable with current diagnostic methods, studies are ongoing to evaluate its usefulness in people with at least 5- to 10-fold increased risk of PDAC. This would include patients with FPC or carriers of a mutation in an established high-penetrance PDAC susceptibility gene (e.g., BRCA2 or PALB2) with at least one case of pancreatic cancer in a first-degree relative (Brand et al., 2007; Bartsch et al., 2012; Klein, 2012; Canto et al., 2013). Furthermore, it has been suggested that such individuals undergo screening for any extrapancreatic tumors associated with their respective germline mutation prior to the development of any respective clinical symptomatology.

USPSTF Screening Guidelines for PDAC have been given a D recommendation indicating harm outweighing any potential benefit and recommending against routine screening in asymptomatic adults using abdominal palpation, ultrasonography, or serologic markers. International Cancer of the Pancreas Screening (CAPS) Consortium summit recommendations for PDAC concluded that screening is recommended for high-risk individuals, although more evidence is needed regarding optimal management of patients with detected lesions. These high-risk candidates for screening include first degree relatives of patients with PDAC from familial kindred with at least two affected first-degree relatives, patients with PJS, and carriers of p16, BRCA2, or HNPCC mutations with at least one affected first-degree relative. The CAPS Consortium was not able to reach a consensus on the age to initiate screening or stop surveillance, as well as screening intervals, although agreement was made that initial screening should include EUS and/or MRI/MRCP, and not CT or ERCP (Canto et al., 2013). At this time, based on the current knowledge of pancreatic susceptibility genes, affected patients of FPC families should consider being tested for the most frequently inherited genetic defects identified in FPC, BRCA2, PALB2, and ATM germline mutations. The use of PDAC biomarkers, such as CA-19-9 and CEACAM-1, have not yet been validated for clinical use in screening (Bussom and Saif, 2010).

To help identify high-risk populations who would be most likely to benefit from early detection screening tests, discovery of additional pancreatic cancer susceptibility genes is crucial (Brentnall et al., 1999; Canto et al., 2006; Koorstra et al., 2008; Vasen et al., 2011; Klein, 2012). Gene expression patterns in serum and tissue biopsies can be studied using whole-genome assaying, including technologies such serial analysis of gene expression (SAGE), cDNA arrays, and oligonucleotide arrays (i.e., gene chips) (Maitra et al., 2006). Further, specific gene-based, gene-product, and marker-based testing for the early detection of pancreatic cancer are currently being developed, which may include miRNAs, which may also be useful as potential therapeutic targets as well (Koorstra et al., 2008).

### Conflict of interest statement

The authors declare that the research was conducted in the absence of any commercial or financial relationships that could be construed as a potential conflict of interest.

## References

[B1] Ardito C. M. Gruner B. M. Takeuchi K. K. Lubeseder-Martellato C. Teichmann N. Mazur P. K. (2012). EGF receptor is required for KRAS-induced pancreatic tumorigenesis. Cancer Cell 22, 304–317 10.1016/j.ccr.2012.07.024 22975374 PMC3443395

[B2] Asano T. Yao Y. Zhu J. Li D. Abbruzzese J. L. Reddy S. A. (2004). The PI 3-kinase/Akt signaling pathway is activated due to aberrant Pten expression and targets transcription factors NF-kappaB and c-Myc in pancreatic cancer cells. Oncogene 23, 8571–8580 10.1038/sj.onc.1207902 15467756

[B3] Asano T. Yao Y. Zhu J. Li D. Abbruzzese J. L. Reddy S. A. (2005). The rapamycin analog CCI-779 is a potent inhibitor of pancreatic cancer cell proliferation. Biochem. Biophys. Res. Commun. 331, 295–302 10.1016/j.bbrc.2005.03.166 15845392

[B4] Axilbund J. E. Argani P. Kamiyama M. Palmisano E. Raben M. Borges M. (2009). Absence of germline BRCA1 mutations in familial pancreatic cancer patients. Cancer Biol. Ther. 8, 131–135 10.4161/cbt.8.2.7136 19029836 PMC2684337

[B5] Barbie D. A. Tamayo P. Boehm J. S. Kim S. Y. Moody S. E. Dunn I. F. (2009). Systematic RNA interference reveals that oncogenic KRAS-driven cancers require TBK1. Nature 462, 108–112 10.1038/nature08460 19847166 PMC2783335

[B6] Bartsch D. K. Gress T. M. Langer P. (2012). Familial pancreatic cancer–current knowledge. Nat. Rev. Gastroenterol. Hepatol. 9, 445–453 10.1038/nrgastro.2012.111 22664588

[B7] Bartsch D. K. Kress R. Sina-Frey M. Grutzmann R. Gerdes B. Pilarsky C. (2004). Prevalence of familial pancreatic cancer in Germany. Int. J. Cancer 110, 902–906 10.1002/ijc.20210 15170674

[B8] Berger N. A. Adams J. W. Sikorski G. W. Petzold S. J. Shearer W. T. (1978). Synthesis of DNA and poly(adenosine diphosphate ribose) in normal and chronic lymphocytic leukemia lymphocytes. J. Clin. Invest. 62, 111–118 10.1172/JCI109094 659624 PMC371743

[B9] Bergmann U. Funatomi H. Yokoyama M. Beger H. G. Korc M. (1995). Insulin-like growth factor I overexpression in human pancreatic cancer: evidence for autocrine and paracrine roles. Cancer Res. 55, 2007–2011 7743492

[B10] Berman D. B. Costalas J. Schultz D. C. Grana G. Daly M. Godwin A. K. (1996). A common mutation in BRCA2 that predisposes to a variety of cancers is found in both Jewish Ashkenazi and non-Jewish individuals. Cancer Res. 56, 3409–3414 8758903

[B11] Berman D. M. Karhadkar S. S. Maitra A. Montes De Oca R. Gerstenblith M. R. Briggs K. (2003). Widespread requirement for Hedgehog ligand stimulation in growth of digestive tract tumours. Nature 425, 846–851 10.1038/nature01972 14520411

[B12] Blackford A. Serrano O. K. Wolfgang C. L. Parmigiani G. Jones S. Zhang X. (2009). SMAD4 gene mutations are associated with poor prognosis in pancreatic cancer. Clin. Cancer Res. 15, 4674–4679 10.1158/1078-0432.CCR-09-0227 19584151 PMC2819274

[B13] Bodoky G. Timcheva C. Spigel D. R. La Stella P. J. Ciuleanu T. E. Pover G. (2012). A phase II open-label randomized study to assess the efficacy and safety of selumetinib (AZD6244 [ARRY-142886]) versus capecitabine in patients with advanced or metastatic pancreatic cancer who have failed first-line gemcitabine therapy. Invest. New Drugs 30, 1216–1223 10.1007/s10637-011-9687-4 21594619

[B14] Brand R. E. Lerch M. M. Rubinstein W. S. Neoptolemos J. P. Whitcomb D. C. Hruban R. H. (2007). Participants of the fourth international symposium of inherited diseases of the, advances in counselling and surveillance of patients at risk for pancreatic cancer. Gut 56, 1460–1469 10.1136/gut.2006.108456 17872573 PMC2000231

[B15] Brand R. E. Lynch H. T. (2006). Genotype/phenotype of familial pancreatic cancer. Endocrinol. Metabol. Clin. N. Am. 35, 405–415 10.1016/j.ecl.2006.02.015 16632102

[B16] Breast Cancer Linkage Consortium (1999). Cancer risks in BRCA2 mutation carriers. J. Natl. Cancer Inst. 91, 1310–1316 10.1093/jnci/91.15.1310 10433620

[B17] Brentnall T. A. Bronner M. P. Byrd D. R. Haggitt R. C. Kimmey M. B. (1999). Early diagnosis and treatment of pancreatic dysplasia in patients with a family history of pancreatic cancer. Ann. Intern. Med. 131, 247–255 10.7326/0003-4819-131-4-199908170-00003 10454945

[B18] Britten C. D. (2013). PI3K and MEK inhibitor combinations: examining the evidence in selected tumor types. Cancer Chemother. Pharmacol. 71, 1395–1409 10.1007/s00280-013-2121-1 23443307

[B19] Brune K. A. Lau B. Palmisano E. Canto M. Goggins M. G. Hruban R. H. (2010). Importance of age of onset in pancreatic cancer kindreds. J. Natl. Cancer Inst. 102, 119–126 10.1093/jnci/djp466 20068195 PMC2808346

[B20] Brunner T. B. Sauer R. Fietkau R. (2011). Gemcitabine/cisplatin versus 5-fluorouracil/mitomycin C chemoradiotherapy in locally advanced pancreatic cancer: a retrospective analysis of 93 patients. Radiat. Oncol. 6:88 10.1186/1748-717X-6-88 21794119 PMC3161863

[B21] Bruns C. J. Koehl G. E. Guba M. Yezhelyev M. Steinbauer M. Seeliger H. (2004). Rapamycin-induced endothelial cell death and tumor vessel thrombosis potentiate cytotoxic therapy against pancreatic cancer. Clin. Cancer Res. 10, 2109–2119 10.1158/1078-0432.CCR-03-0502 15041732

[B22] Bruns C. J. Solorzano C. C. Harbison M. T. Ozawa S. Tsan R. Fan D. (2000). Blockade of the epidermal growth factor receptor signaling by a novel tyrosine kinase inhibitor leads to apoptosis of endothelial cells and therapy of human pancreatic carcinoma. Cancer Res. 60, 2926–2935 10850439

[B23] Bryant H. E. Schultz N. Thomas H. D. Parker K. M. Flower D. Lopez E. (2005). Specific killing of BRCA2-deficient tumours with inhibitors of poly(ADP-ribose) polymerase. Nature 434, 913–917 10.1038/nature03443 15829966

[B24] Bussom S. Saif M. W. (2010). Methods and rationale for the early detection of pancreatic cancer. Highlights from the “2010 ASCO Gastrointestinal Cancers Symposium.” Orlando, FL. January 22–24, 2010. JOP 11, 128–130 20208319

[B25] Caldas C. Hahn S. A. da Costa L. T. Redston M. S. Schutte M. Seymour A. B. (1994). Frequent somatic mutations and homozygous deletions of the p16 (MTS1) gene in pancreatic adenocarcinoma. Nat. Genet. 8, 27–32 10.1038/ng0994-27 7726912

[B26] Calhoun E. S. Jones J. B. Ashfaq R. Adsay V. Baker S. J. Valentine V. (2003). BRAF and FBXW7 (CDC4, FBW7, AGO, SEL10) mutations in distinct subsets of pancreatic cancer: potential therapeutic targets. Am. J. Pathol. 163, 1255–1260 10.1016/S0002-9440(10)63485-2 14507635 PMC1868306

[B27] Canto M. I. Goggins M. Hruban R. H. Petersen G. M. Giardiello F. M. Yeo C. (2006). Screening for early pancreatic neoplasia in high-risk individuals: a prospective controlled study. Clin. Gastroenterol. Hepatol. 4, 766–781, quiz: 665. 10.1016/j.cgh.2006.02.005 16682259

[B28] Canto M. I. Harinck F. Hruban R. H. Offerhaus G. J. Poley J. W. Kamel I. (2013). International cancer of the pancreas screening (CAPS) Consortium summit on the management of patients with increased risk for familial pancreatic cancer. Gut 62, 339–347 10.1136/gutjnl-2012-303108 23135763 PMC3585492

[B29] Carboni J. M. Wittman M. Yang Z. Lee F. Greer A. Hurlburt W. (2009). BMS-754807, a small molecule inhibitor of insulin-like growth factor-1R/IR. Mol. Cancer Ther. 8, 3341–3349 10.1158/1535-7163.MCT-09-0499 19996272

[B30] Cereda S. Reni M. Rognone A. Fugazza C. Ghidini M. Ceraulo D. (2011). Salvage therapy with mitomycin and ifosfamide in patients with gemcitabine-resistant metastatic pancreatic cancer: a phase II trial. Chemotherapy 57, 156–161 10.1159/000324865 21454973

[B31] Cheng J. Q. Ruggeri B. Klein W. M. Sonoda G. Altomare D. A. Watson D. K. (1996). Amplification of AKT2 in human pancreatic cells and inhibition of AKT2 expression and tumorigenicity by antisense RNA. Proc. Natl. Acad. Sci. U.S.A. 93, 3636–3641 10.1073/pnas.93.8.3636 8622988 PMC39663

[B32] Couch F. J. Johnson M. R. Rabe K. G. Brune K. de Andrade M. Goggins M. (2007). The prevalence of BRCA2 mutations in familial pancreatic cancer. Cancer Epidemiol. Biomarkers Prev. 16, 342–346 10.1158/1055-9965.EPI-06-0783 17301269

[B33] Crane C. H. Ellis L. M. Abbruzzese J. L. Amos C. Xiong H. Q. Ho L. (2006). Phase I trial evaluating the safety of bevacizumab with concurrent radiotherapy and capecitabine in locally advanced pancreatic cancer. J. Clin. Oncol. 24, 1145–1151 10.1200/JCO.2005.03.6780 16505434

[B34] Crane C. H. Varadhachary G. R. Yordy J. S. Staerkel G. A. Javle M. M. Safran H. (2011). Phase II trial of cetuximab, gemcitabine, and oxaliplatin followed by chemoradiation with cetuximab for locally advanced (T4) pancreatic adenocarcinoma: correlation of Smad4(Dpc4) immunostaining with pattern of disease progression. J. Clin. Oncol. 29, 3037–3043 10.1200/JCO.2010.33.8038 21709185 PMC3157965

[B35] da Cunha Santos G. Dhani N. Tu D. Chin K. Ludkovski O. Kamel-Reid S. (2010). Molecular predictors of outcome in a phase 3 study of gemcitabine and erlotinib therapy in patients with advanced pancreatic cancer: national cancer institute of Canada clinical trials group study PA.3. Cancer 116, 5599–5607 10.1002/cncr.25393 20824720

[B36] Del Chiaro M. Zerbi A. Falconi M. Bertacca L. Polese M. Sartori N. (2007). Cancer risk among the relatives of patients with pancreatic ductal adenocarcinoma. Pancreatology 7, 459–469 10.1159/000108963 17912010

[B37] Derynck R. Zhang Y. E. (2003). Smad-dependent and Smad-independent pathways in TGF-beta family signalling. Nature 425, 577–584 10.1038/nature02006 14534577

[B38] di Magliano M. P. Logsdon C. D. (2013). Roles for KRAS in pancreatic tumor development and progression. Gastroenterology 144, 1220–1229 10.1053/j.gastro.2013.01.071 23622131 PMC3902845

[B39] Ducreux M. Boige V. Goere D. Deutsch E. Ezra P. Elias D. (2007). The multidisciplinary management of gastrointestinal cancer. Pancreatic cancer: from pathogenesis to cure. Best Pract. Res. Clin.Gastroenterol. 21, 997–1014 10.1016/j.bpg.2007.10.025 18070700

[B40] Ehrenthal D. Haeger L. Griffin T. Compton C. (1987). Familial pancreatic adenocarcinoma in three generations. A case report and a review of the literature. Cancer 59, 1661–1664 10.1002/1097-0142(19870501)59:9<1661::AID-CNCR2820590923>3.0.CO;2-H 3828965

[B41] Engelman J. A. Chen L. Tan X. Crosby K. Guimaraes A. R. Upadhyay R. (2008). Effective use of PI3K and MEK inhibitors to treat mutant Kras G12D and PIK3CA H1047R murine lung cancers. Nat. Med. 14, 1351–1356 10.1038/nm.1890 19029981 PMC2683415

[B42] Farmer H. McCabe N. Lord C. J. Tutt A. N. Johnson D. A. Richardson T. B. (2005). Targeting the DNA repair defect in BRCA mutant cells as a therapeutic strategy. Nature 434, 917–921 10.1038/nature03445 15829967

[B43] Feldmann G. Karikari C. dal Molin M. Duringer S. Volkmann P. Bartsch D. K. (2011). Inactivation of Brca2 cooperates with Trp53(R172H) to induce invasive pancreatic ductal adenocarcinomas in mice: a mouse model of familial pancreatic cancer. Cancer Biol. Ther. 11, 959–968 10.4161/cbt.11.11.15534 21455033 PMC3127047

[B45] Ferrone C. R. Levine D. A. Tang L. H. Allen P. J. Jarnagin W. Brennan M. F. (2009). BRCA germline mutations in Jewish patients with pancreatic adenocarcinoma. J. Clin. Oncol. 27, 433–438 10.1200/JCO.2008.18.5546 19064968 PMC3657622

[B46] Friess H. Berberat P. Schilling M. Kunz J. Korc M. Buchler M. W. (1996). Pancreatic cancer: the potential clinical relevance of alterations in growth factors and their receptors. J. Mol. Med. 74, 35–42 10.1007/BF00202070 8834768

[B47] Ghadirian P. Boyle P. Simard A. Baillargeon J. Maisonneuve P. Perret C. (1991). Reported family aggregation of pancreatic cancer within a population-based case-control study in the Francophone community in Montreal, Canada. Int. J. Pancreatol. 10, 183–196 1787333 10.1007/BF02924156

[B48] Giardiello F. M. Brensinger J. D. Tersmette A. C. Goodman S. N. Petersen G. M. Booker S. V. (2000). Very high risk of cancer in familial Peutz-Jeghers syndrome. Gastroenterology 119, 1447–1453 10.1053/gast.2000.20228 11113065

[B49] Goggins M. Hruban R. H. Kern S. E. (2000). BRCA2 is inactivated late in the development of pancreatic intraepithelial neoplasia: evidence and implications. Am. J. Pathol. 156, 1767–1771 10.1016/S0002-9440(10)65047-X 10793087 PMC1876938

[B50] Goggins M. Schutte M. Lu J. Moskaluk C. A. Weinstein C. L. Petersen G. M. (1996). Germline BRCA2 gene mutations in patients with apparently sporadic pancreatic carcinomas. Cancer Res. 56, 5360–5364 8968085

[B51] Gruis N. A. van der Velden P. A. Sandkuijl L. A. Prins D. E. Weaver-Feldhaus J. Kamb A. (1995). Homozygotes for CDKN2 (p16) germline mutation in Dutch familial melanoma kindreds. Nat. Genet. 10, 351–353 10.1038/ng0795-351 7670475

[B52] Hahn S. A. Greenhalf B. Ellis I. Sina-Frey M. Rieder H. Korte B. (2003). BRCA2 germline mutations in familial pancreatic carcinoma. J. Natl. Cancer Inst. 95, 214–221 10.1093/jnci/95.3.214 12569143

[B53] Hahn S. A. Schmiegel W. H. (1998). Recent discoveries in cancer genetics of exocrine pancreatic neoplasia. Digestion 59, 493–501 10.1159/000007526 9705534

[B54] Hakam A. Fang Q. Karl R. Coppola D. (2003). Coexpression of IGF-1R and c-Src proteins in human pancreatic ductal adenocarcinoma. Dig. Dis. Sci. 48, 1972–1978 10.1023/A:1026122421369 14627343

[B55] Hemminki K. Li X. (2003). Familial and second primary pancreatic cancers: a nationwide epidemiologic study from Sweden. Int. J. Cancer 103, 525–530 10.1002/ijc.10863 12478670

[B56] Hewish M. Chau I. Cunningham D. (2009). Insulin-like growth factor 1 receptor targeted therapeutics: novel compounds and novel treatment strategies for cancer medicine. Recent Pat. Anticancer Drug Discov. 4, 54–72 10.2174/157489209787002515 19149688

[B57] Hezel A. F. Kimmelman A. C. Stanger B. Z. Bardeesy N. Depinho R. A. (2006). Genetics and biology of pancreatic ductal adenocarcinoma. Genes Dev. 20, 1218–1249 10.1101/gad.1415606 16702400

[B58] Hruban R. H. Canto M. I. Goggins M. Schulick R. Klein A. P. (2010). Update on familial pancreatic cancer. Adv. Surg. 44, 293–311 10.1016/j.yasu.2010.05.011 20919528 PMC2966038

[B59] Hruban R. H. Goggins M. Parsons J. Kern S. E. (2000). Progression model for pancreatic cancer. Clin. Cancer Res. 6, 2969–2972 10955772

[B60] Hruban R. H. Petersen G. M. Goggins M. Tersmette A. C. Offerhaus G. J. Falatko F. (1999). Familial pancreatic cancer. Ann. Oncol. 10(Suppl. 4), 69–73 10.1093/annonc/10.suppl_4.S69 10436789

[B61] Hruban R. H. Petersen G. M. Ha P. K. Kern S. E. (1998). Genetics of pancreatic cancer. From genes to families. Surg. Oncol. Clin. N. Am. 7, 1–23 9443984

[B62] Hruban R. H. van Mansfeld A. D. Offerhaus G. J. van Weering D. H. Allison D. C. Goodman S. N. (1993). K-ras oncogene activation in adenocarcinoma of the human pancreas. A study of 82 carcinomas using a combination of mutant-enriched polymerase chain reaction analysis and allele-specific oligonucleotide hybridization. Am. J. Pathol. 143, 545–554 8342602 PMC1887038

[B63] Hustinx S. R. Hruban R. H. Leoni L. M. Iacobuzio-Donahue C. Cameron J. L. Yeo C. J. (2005). Homozygous deletion of the MTAP gene in invasive adenocarcinoma of the pancreas and in periampullary cancer: a potential new target for therapy. Cancer Biol. Ther. 4, 83–86 10.4161/cbt.4.1.1380 15662124

[B64] Iacobuzio-Donahue C. A. Fu B. Yachida S. Luo M. Abe H. Henderson C. M. (2009). DPC4 gene status of the primary carcinoma correlates with patterns of failure in patients with pancreatic cancer. J. Clin. Oncol. 27, 1806–1813 10.1200/JCO.2008.17.7188 19273710 PMC2668706

[B65] Jacobs E. J. Chanock S. J. Fuchs C. S. Lacroix A. McWilliams R. R. Steplowski E. (2010). Family history of cancer and risk of pancreatic cancer: a pooled analysis from the Pancreatic Cancer Cohort Consortium (PanScan). Int. J. Cancer 127, 1421–1428 10.1002/ijc.25148 20049842 PMC2926939

[B66] James G. L. Goldstein J. L. Brown M. S. Rawson T. E. Somers T. C. McDowell R. S. (1993). Benzodiazepine peptidomimetics: potent inhibitors of Ras farnesylation in animal cells. Science 260, 1937–1942 10.1126/science.8316834 8316834

[B67] James T. A. Sheldon D. G. Rajput A. Kuvshinoff B. W. Javle M. M. Nava H. R. (2004). Risk factors associated with earlier age of onset in familial pancreatic carcinoma. Cancer 101, 2722–2726 10.1002/cncr.20700 15534880

[B68] Jenne D. E. Reimann H. Nezu J. Friedel W. Loff S. Jeschke R. (1998). Peutz-Jeghers syndrome is caused by mutations in a novel serine threonine kinase. Nat. Genet. 18, 38–43 10.1038/ng0198-38 9425897

[B69] Jones S. Hruban R. H. Kamiyama M. Borges M. Zhang X. Parsons D. W. (2009). Exomic sequencing identifies PALB2 as a pancreatic cancer susceptibility gene. Science 324, 217 10.1126/science.1171202 19264984 PMC2684332

[B70] Jones S. Zhang X. Parsons D. W. Lin J. C. Leary R. J. Angenendt P. (2008). Core signaling pathways in human pancreatic cancers revealed by global genomic analyses. Science 321, 1801–1806 10.1126/science.1164368 18772397 PMC2848990

[B71] Kelleher F. C. (2011). Hedgehog signaling and therapeutics in pancreatic cancer. Carcinogenesis 32, 445–451 10.1093/carcin/bgq280 21186299

[B72] Kindler H. L. Friberg G. Singh D. A. Locker G. Nattam S. Kozloff M. (2005). Phase II trial of bevacizumab plus gemcitabine in patients with advanced pancreatic cancer. J. Clin. Oncol. 23, 8033–8040 10.1200/JCO.2005.01.9661 16258101

[B73] Kindler H. L. Niedzwiecki D. Hollis D. Sutherland S. Schrag D. Hurwitz H. (2010). Gemcitabine plus bevacizumab compared with gemcitabine plus placebo in patients with advanced pancreatic cancer: phase III trial of the Cancer and Leukemia Group B (CALGB 80303). J. Clin. Oncol. 28, 3617–3622 10.1200/JCO.2010.28.1386 20606091 PMC2917317

[B74] Kindler H. L. Richards D. A. Garbo L. E. Garon E. B. Stephenson J. J. Jr., Rocha-Lima C. M. (2012). randomized, A, placebo-controlled phase 2 study of ganitumab (AMG 479) or conatumumab (AMG 655) in combination with gemcitabine in patients with metastatic pancreatic cancer. Ann. Oncol. 23, 2834–2842 10.1093/annonc/mds142 22700995

[B75] Klein A. P. (2012). Genetic susceptibility to pancreatic cancer. Mol. Carcinog. 51, 14–24 10.1002/mc.20855 22162228 PMC3570154

[B76] Klein A. P. Brune K. A. Petersen G. M. Goggins M. Tersmette A. C. Offerhaus G. J. (2004). Prospective risk of pancreatic cancer in familial pancreatic cancer kindreds. Cancer Res. 64, 2634–2638 10.1158/0008-5472.CAN-03-3823 15059921

[B77] Klein A. P. Hruban R. H. Brune K. A. Petersen G. M. Goggins M. (2001). Familial pancreatic cancer. Cancer J. 7, 266–273 11561603

[B78] Koorstra J. B. Hustinx S. R. Offerhaus G. J. Maitra A. (2008). Pancreatic carcinogenesis. Pancreatology 8, 110–125 10.1159/000123838 18382097 PMC2663569

[B79] Kraakman-van der Zwet M. Overkamp W. J. van Lange R. E. Essers J. van Duijn-Goedhart A. Wiggers I. (2002). Brca2 (XRCC11) deficiency results in radioresistant DNA synthesis and a higher frequency of spontaneous deletions. Mol. Cell. Biol. 22, 669–679 10.1128/MCB.22.2.669-679.2002 11756561 PMC139737

[B80] Krainer M. Silva-Arrieta S. FitzGerald M. G. Shimada A. Ishioka C. Kanamaru R. (1997). Differential contributions of BRCA1 and BRCA2 to early-onset breast cancer. N. Engl. J. Med. 336, 1416–1421 10.1056/NEJM199705153362003 9145678

[B81] Lal G. Liu G. Schmocker B. Kaurah P. Ozcelik H. Narod S. A. (2000). Inherited predisposition to pancreatic adenocarcinoma: role of family history and germ-line p16, BRCA1, and BRCA2 mutations. Cancer Res. 60, 409–416 10667595

[B82] Langston A. A. Malone K. E. Thompson J. D. Daling J. R. Ostrander E. A. (1996). BRCA1 mutations in a population-based sample of young women with breast cancer. N. Engl. J. Med. 334, 137–142 10.1056/NEJM199601183340301 8531967

[B83] Li D. Xie K. Wolff R. Abbruzzese J. L. (2004a). Pancreatic cancer. Lancet 363, 1049–1057 10.1016/S0140-6736(04)15841-8 15051286

[B84] Li J. Kleeff J. Giese N. Buchler M. W. Korc M. Friess H. (2004b). Gefitinib (‘Iressa’, ZD1839), a selective epidermal growth factor receptor tyrosine kinase inhibitor, inhibits pancreatic cancer cell growth, invasion, and colony formation. Int. J. Oncol. 25, 203–210 15202007

[B85] Lorusso P. M. Adjei A. A. Varterasian M. Gadgeel S. Reid J. Mitchell D. Y. (2005). Phase I and pharmacodynamic study of the oral MEK inhibitor CI-1040 in patients with advanced malignancies. J. Clin. Oncol. 23, 5281–5293 10.1200/JCO.2005.14.415 16009947

[B86] LoRusso P. M. Rudin C. M. Reddy J. C. Tibes R. Weiss G. J. Borad M. J. (2011). Phase I trial of hedgehog pathway inhibitor vismodegib (GDC-0449) in patients with refractory, locally advanced or metastatic solid tumors. Clin. Cancer Res. 17, 2502–2511 10.1158/1078-0432.CCR-10-2745 21300762 PMC5244484

[B87] Lu X. Xu T. Qian J. Wen X. Wu D. (2002). Detecting K-ras and p53 gene mutation from stool and pancreatic juice for diagnosis of early pancreatic cancer. Chin. Med. J. 115, 1632–1636 12609076

[B88] Lubinski J. Phelan C. M. Ghadirian P. Lynch H. T. Garber J. Weber B. (2004). Cancer variation associated with the position of the mutation in the BRCA2 gene. Fam. Cancer 3, 1–10 10.1023/B:FAME.0000026816.32400.45 15131399

[B89] Lynch H. T. Fitzsimmons M. L. Smyrk T. C. Lanspa S. J. Watson P. McClellan J. (1990). Familial pancreatic cancer: clinicopathologic study of 18 nuclear families. Am. J. Gastroenterol. 85, 54–60 2296965

[B90] Lynch H. T. Fusaro R. M. Lynch J. F. Brand R. (2008). Pancreatic cancer and the FAMMM syndrome. Fam. Cancer 7, 103–112 10.1007/s10689-007-9166-4 17992582

[B91] Lynch H. T. Smyrk T. Kern S. E. Hruban R. H. Lightdale C. J. Lemon S. J. (1996). Familial pancreatic cancer: a review. Semin. Oncol. 23, 251–275 8623061

[B92] MacDermott R. P. Kramer P. (1973). Adenocarcinoma of the pancreas in four siblings. Gastroenterology 65, 137–139 4720820

[B93] Maitra A. Kern S. E. Hruban R. H. (2006). Molecular pathogenesis of pancreatic cancer. Best Pract. Res. Clin. Gastroenterol. 20, 211–226 10.1016/j.bpg.2005.10.002 16549325

[B94] Maloney E. K. McLaughlin J. L. Dagdigian N. E. Garrett L. M. Connors K. M. Zhou X. M. (2003). An anti-insulin-like growth factor I receptor antibody that is a potent inhibitor of cancer cell proliferation. Cancer Res. 63, 5073–5083 12941837

[B95] McFaul C. D. Greenhalf W. Earl J. Howes N. Neoptolemos J. P. Kress R. (2006). Anticipation in familial pancreatic cancer. Gut 55, 252–258 10.1136/gut.2005.065045 15972300 PMC1856528

[B96] Mimeault M. Batra S. K. (2010). Frequent deregulations in the hedgehog signaling network and cross-talks with the epidermal growth factor receptor pathway involved in cancer progression and targeted therapies. Pharmacol. Rev. 62, 497–524 10.1124/pr.109.002329 20716670 PMC2964899

[B97] Min Y. Adachi Y. Yamamoto H. Ito H. Itoh F. Lee C. T. (2003). Genetic blockade of the insulin-like growth factor-I receptor: a promising strategy for human pancreatic cancer. Cancer Res. 63, 6432–6441 14559833

[B98] Moore M. J. Goldstein D. Hamm J. Figer A. Hecht J. R. Gallinger S. (2007). Erlotinib plus gemcitabine compared with gemcitabine alone in patients with advanced pancreatic cancer: a phase III trial of the National Cancer Institute of Canada Clinical Trials Group. J. Clin. Oncol. 25, 1960–1966 10.1200/JCO.2006.07.9525 17452677

[B99] Moran A. O'Hara C. Khan S. Shack L. Woodward E. Maher E. R. (2012). Risk of cancer other than breast or ovarian in individuals with BRCA1 and BRCA2 mutations. Fam. Cancer 11, 235–242 10.1007/s10689-011-9506-2 22187320

[B100] Murphy K. M. Brune K. A. Griffin C. Sollenberger J. E. Petersen G. M. Bansal R. (2002). Evaluation of candidate genes MAP2K4, MADH4, ACVR1B, and BRCA2 in familial pancreatic cancer: deleterious BRCA2 mutations in 17%. Cancer Res. 62, 3789–3793 12097290

[B101] Nair P. N. De Armond D. T. Adamo M. L. Strodel W. E. Freeman J. W. (2001). Aberrant expression and activation of insulin-like growth factor-1 receptor (IGF-1R) are mediated by an induction of IGF-1R promoter activity and stabilization of IGF-1R mRNA and contributes to growth factor independence and increased survival of the pancreatic cancer cell line MIA PaCa-2. Oncogene 20, 8203–8214 10.1038/sj.onc.1205044 11781836

[B102] Oddoux C. Struewing J. P. Clayton C. M. Neuhausen S. Brody L. C. Kaback M. (1996). The carrier frequency of the BRCA2 6174delT mutation among Ashkenazi Jewish individuals is approximately 1%. Nat. Genet. 14, 188–190 10.1038/ng1096-188 8841192

[B103] Olive K. P. Jacobetz M. A. Davidson C. J. Gopinathan A. McIntyre D. Honess D. (2009). Inhibition of Hedgehog signaling enhances delivery of chemotherapy in a mouse model of pancreatic cancer. Science 324, 1457–1461 10.1126/science.1171362 19460966 PMC2998180

[B104] Ouban A. Muraca P. Yeatman T. Coppola D. (2003). Expression and distribution of insulin-like growth factor-1 receptor in human carcinomas. Hum. Pathol. 34, 803–808 10.1016/S0046-8177(03)00291-0 14506643

[B105] Ozcelik H. Schmocker B. Di Nicola N. Shi X. H. Langer B. Moore M. (1997). Germline BRCA2 6174delT mutations in Ashkenazi Jewish pancreatic cancer patients. Nat. Genet. 16, 17–18 10.1038/ng0597-17 9140390

[B106] Pasca di Magliano M. Sekine S. Ermilov A. Ferris J. Dlugosz A. A. Hebrok M. (2006). Hedgehog/Ras interactions regulate early stages of pancreatic cancer. Genes Dev. 20, 3161–3173 10.1101/gad.1470806 17114586 PMC1635150

[B107] Patel K. J. Yu V. P. Lee H. Corcoran A. Thistlethwaite F. C. Evans M. J. (1998). Involvement of Brca2 in DNA repair. Mol. Cell 1, 347–357 10.1016/S1097-2765(00)80035-0 9660919

[B108] Permuth-Wey J. Egan K. M. (2009). Family history is a significant risk factor for pancreatic cancer: results from a systematic review and meta-analysis. Fam. Cancer 8, 109–117 10.1007/s10689-008-9214-8 18763055

[B109] Phelan C. M. Lancaster J. M. Tonin P. Gumbs C. Cochran C. Carter R. (1996). Mutation analysis of the BRCA2 gene in 49 site-specific breast cancer families. Nat. Genet. 13, 120–122 10.1038/ng0596-120 8673090

[B110] Philip P. A. Benedetti J. Corless C. L. Wong R. O'Reilly E. M. Flynn P. J. (2010). Phase III study comparing gemcitabine plus cetuximab versus gemcitabine in patients with advanced pancreatic adenocarcinoma: southwest oncology group-directed intergroup trial S(0205). J. Clin. Oncol. 28, 3605–3610 10.1200/JCO.2009.25.7550 20606093 PMC2917315

[B111] Rahman N. Seal S. Thompson D. Kelly P. Renwick A. Elliott A. (2007). PALB2, which encodes a BRCA2-interacting protein, is a breast cancer susceptibility gene. Nat. Genet. 39, 165–167 10.1038/ng1959 17200668 PMC2871593

[B112] Redston M. S. Caldas C. Seymour A. B. Hruban R. H. da Costa L. Yeo C. J. (1994). p53 mutations in pancreatic carcinoma and evidence of common involvement of homocopolymer tracts in DNA microdeletions. Cancer Res. 54, 3025–3033 8187092

[B113] Reichert M. Saur D. Hamacher R. Schmid R. M. Schneider G. (2007). Phosphoinositide-3-kinase signaling controls S-phase kinase-associated protein 2 transcription via E2F1 in pancreatic ductal adenocarcinoma cells. Cancer Res. 67, 4149–4156 10.1158/0008-5472.CAN-06-4484 17483325

[B114] Reimer R. R. Fraumeni J. F. Jr., Ozols R. F. Bender R. (1977). Pancreatic cancer in father and son. Lancet 1, 911 10.1016/S0140-6736(77)91244-2 67325

[B115] Rinehart J. Adjei A. A. Lorusso P. M. Waterhouse D. Hecht J. R. Natale R. B. (2004). Multicenter phase II study of the oral inhibitor, MEK, CI-1040, in patients with advanced non-small-cell lung, breast, colon, and pancreatic cancer. J. Clin. Oncol. 22, 4456–4462 10.1200/JCO.2004.01.185 15483017

[B116] Rowley M. Ohashi A. Mondal G. Mills L. Yang L. Zhang L. (2011). Inactivation of Brca2 promotes Trp53-associated but inhibits KrasG12D-dependent pancreatic cancer development in mice. Gastroenterology 140, 1303–1313e1–e3. 10.1053/j.gastro.2010.12.039 21199651 PMC3066280

[B117] Rozenblum E. Schutte M. Goggins M. Hahn S. A. Panzer S. Zahurak M. (1997). Tumor-suppressive pathways in pancreatic carcinoma. Cancer Res. 57, 1731–1734 9135016

[B118] Ruggeri B. A. Huang L. Wood M. Cheng J. Q. Testa J. R. (1998). Amplification and overexpression of the AKT2 oncogene in a subset of human pancreatic ductal adenocarcinomas. Mol. Carcinog. 21, 81–86 10.1002/(SICI)1098-2744(199802)21:2<81::AID-MC1>3.0.CO;2-R 9496907

[B119] Rulyak S. J. Lowenfels A. B. Maisonneuve P. Brentnall T. A. (2003). Risk factors for the development of pancreatic cancer in familial pancreatic cancer kindreds. Gastroenterology 124, 1292–1299 10.1016/S0016-5085(03)00272-5 12730869

[B120] Sato N. Rosty C. Jansen M. Fukushima N. Ueki T. Yeo C. J. (2001). STK11/LKB1 Peutz-Jeghers gene inactivation in intraductal papillary-mucinous neoplasms of the pancreas. Am. J. Pathol. 159, 2017–2022 10.1016/S0002-9440(10)63053-2 11733352 PMC1850608

[B121] Schenk M. Schwartz A. G. O'Neal E. Kinnard M. Greenson J. K. Fryzek J. P. (2001). Familial risk of pancreatic cancer. J. Natl. Cancer Inst. 93, 640–644 10.1093/jnci/93.8.640 11309441

[B122] Schlieman M. G. Fahy B. N. Ramsamooj R. Beckett L. Bold R. J. (2003). Incidence, mechanism and prognostic value of activated AKT in pancreas cancer. Br. J. Cancer 89, 2110–2115 10.1038/sj.bjc.6601396 14647146 PMC2376856

[B123] Schnall S. F. Macdonald J. S. (1996). Chemotherapy of adenocarcinoma of the pancreas. Semin. Oncol. 23, 220–228 8623058

[B124] Schneider G. Schmid R. M. (2003). Genetic alterations in pancreatic carcinoma. Mol. Cancer 2:15 10.1186/1476-4598-2-15 12605716 PMC150381

[B125] Schneider R. Slater E. P. Sina M. Habbe N. Fendrich V. Matthai E. (2011). German national case collection for familial pancreatic cancer (FaPaCa): ten years experience. Fam. Cancer 10, 323–330 10.1007/s10689-010-9414-x 21207249

[B126] Schonleben F. Qiu W. Ciau N. T. Ho D. J. Li X. Allendorf J. D. (2006). PIK3CA mutations in intraductal papillary mucinous neoplasm/carcinoma of the pancreas. Clin. Cancer Res. 12, 3851–3855 10.1158/1078-0432.CCR-06-0292 16778113 PMC1780026

[B127] Sharan S. K. Morimatsu M. Albrecht U. Lim D. S. Regel E. Dinh C. (1997). Embryonic lethality and radiation hypersensitivity mediated by Rad51 in mice lacking Brca2. Nature 386, 804–810 10.1038/386804a0 9126738

[B128] Siegel R. Naishadham D. Jemal A. (2013). Cancer statistics (2013). CA Cancer J. Clin. 63, 11–30 10.3322/caac.21166 23335087

[B129] Silverman D. T. Schiffman M. Everhart J. Goldstein A. Lillemoe K. D. Swanson G. M. (1999). Diabetes mellitus, other medical conditions and familial history of cancer as risk factors for pancreatic cancer. Br. J. Cancer 80, 1830–1837 10.1038/sj.bjc.6690607 10468306 PMC2363127

[B130] Skoulidis F. Cassidy L. D. Pisupati V. Jonasson J. G. Bjarnason H. Eyfjord J. E. (2010). Germline Brca2 heterozygosity promotes Kras(G12D) -driven carcinogenesis in a murine model of familial pancreatic cancer. Cancer Cell 18, 499–509 10.1016/j.ccr.2010.10.015 21056012

[B131] Slater E. P. Langer P. Niemczyk E. Strauch K. Butler J. Habbe N. (2010). PALB2 mutations in European familial pancreatic cancer families. Clin. Genet. 78, 490–494 10.1111/j.1399-0004.2010.01425.x 20412113

[B132] Spano J. P. Chodkiewicz C. Maurel J. Wong R. Wasan H. Barone C. (2008). Efficacy of gemcitabine plus axitinib compared with gemcitabine alone in patients with advanced pancreatic cancer: an open-label randomised phase II study. Lancet 371, 2101–2108 10.1016/S0140-6736(08)60661-3 18514303

[B133] Stoecklein N. H. Luebke A. M. Erbersdobler A. Knoefel W. T. Schraut W. Verde P. E. (2004). Copy number of chromosome 17 but not HER2 amplification predicts clinical outcome of patients with pancreatic ductal adenocarcinoma. J. Clin. Oncol. 22, 4737–4745 10.1200/JCO.2004.05.142 15570074

[B134] Stoeltzing O. Liu W. Reinmuth N. Fan F. Parikh A. A. Bucana C. D. (2003). Regulation of hypoxia-inducible factor-1alpha, vascular endothelial growth factor, and angiogenesis by an insulin-like growth factor-I receptor autocrine loop in human pancreatic cancer. Am. J. Pathol. 163, 1001–1011 10.1016/S0002-9440(10)63460-8 12937141 PMC1868239

[B135] Talar-Wojnarowska R. Malecka-Panas E. (2006). Molecular pathogenesis of pancreatic adenocarcinoma: potential clinical implications. Med. Sci. Monit. 12, RA186–RA193 16940943

[B136] Tanno S. Tanno S. Mitsuuchi Y. Altomare D. A. Xiao G. H. Testa J. R. (2001). AKT activation up-regulates insulin-like growth factor I receptor expression and promotes invasiveness of human pancreatic cancer cells. Cancer Res. 61, 589–593 11212254

[B137] Tersmette A. C. Petersen G. M. Offerhaus G. J. Falatko F. C. Brune K. A. Goggins M. (2001). Increased risk of incident pancreatic cancer among first-degree relatives of patients with familial pancreatic cancer. Clin. Cancer Res. 7, 738–744 11297271

[B138] Thompson D. Easton D. F. Breast Cancer Linkage Consortium (2002). Cancer incidence in BRCA1 mutation carriers. J. Natl. Cancer Inst. 94, 1358–1365 10.1093/jnci/94.18.1358 12237281

[B139] Tischkowitz M. D. Sabbaghian N. Hamel N. Borgida A. Rosner C. Taherian N. (2009). Analysis of the gene coding for the BRCA2-interacting protein PALB2 in familial and sporadic pancreatic cancer. Gastroenterology 137, 1183–1186 10.1053/j.gastro.2009.06.055 19635604 PMC3914669

[B140] van Asperen C. J. Brohet R. M. Meijers-Heijboer E. J. Hoogerbrugge N. Verhoef S. Vasen H. F. (2005). Cancer risks in BRCA2 families: estimates for sites other than breast and ovary. J. Med. Genet. 42, 711–719 10.1136/jmg.2004.028829 16141007 PMC1736136

[B141] Van Cutsem E. van de Velde H. Karasek P. Oettle H. Vervenne W. L. Szawlowski A. (2004). Phase III trial of gemcitabine plus tipifarnib compared with gemcitabine plus placebo in advanced pancreatic cancer. J. Clin. Oncol. 22, 1430–1438 10.1200/JCO.2004.10.112 15084616

[B142] van der Heijden M. S. Brody J. R. Dezentje D. A. Gallmeier E. Cunningham S. C. Swartz M. J. (2005). *In vivo* therapeutic responses contingent on Fanconi anemia/BRCA2 status of the tumor. Clin. Cancer Res. 11, 7508–7515 10.1158/1078-0432.CCR-05-1048 16243825

[B143] Vasen H. F. Wasser M. van Mil A. Tollenaar R. A. Konstantinovski M. Gruis N. A. (2011). Magnetic resonance imaging surveillance detects early-stage pancreatic cancer in carriers of a p16-Leiden mutation. Gastroenterology 140, 850–856 10.1053/j.gastro.2010.11.048 21129377

[B144] Wang W. Chen S. Brune K. A. Hruban R. H. Parmigiani G. Klein A. P. (2007). PancPRO: risk assessment for individuals with a family history of pancreatic cancer. J. Clin. Oncol. 25, 1417–1422 10.1200/JCO.2006.09.2452 17416862 PMC2267288

[B145] Wilentz R. E. Su G. H. Dai J. L. Sparks A. B. Argani P. Sohn T. A. (2000). Immunohistochemical labeling for dpc4 mirrors genetic status in pancreatic adenocarcinomas : a new marker of DPC4 inactivation. Am. J. Pathol. 156, 37–43 10.1016/S0002-9440(10)64703-7 10623651 PMC1868651

[B146] Willett C. G. Kozin S. V. Duda D. G. di Tomaso E. Kozak K. R. Boucher Y. (2006). Combined vascular endothelial growth factor-targeted therapy and radiotherapy for rectal cancer: theory and clinical practice. Semin. Oncol. 33, S35–S40 10.1053/j.seminoncol.2006.08.007 17145523 PMC2686124

[B147] Williamson C. T. Kubota E. Hamill J. D. Klimowicz A. Ye R. Muzik H. (2012). Enhanced cytotoxicity of PARP inhibition in mantle cell lymphoma harbouring mutations in both ATM and p53. EMBO Mol. Med. 4, 515–527 10.1002/emmm.201200229 22416035 PMC3443945

[B148] Wolpin B. M. Hezel A. F. Abrams T. Blaszkowsky L. S. Meyerhardt J. A. Chan J. A. (2009). Oral mTOR inhibitor everolimus in patients with gemcitabine-refractory metastatic pancreatic cancer. J. Clin. Oncol. 27, 193–198 10.1200/JCO.2008.18.9514 19047305 PMC2645085

[B149] Xia B. Sheng Q. Nakanishi K. Ohashi A. Wu J. Christ N. (2006). Control of BRCA2 cellular and clinical functions by a nuclear partner, PALB2. Mol. Cell 22, 719–729 10.1016/j.molcel.2006.05.022 16793542

[B150] Xin W. Yun K. J. Ricci F. Zahurak M. Qiu W. Su G. H. (2004). MAP2K4/MKK4 expression in pancreatic cancer: genetic validation of immunohistochemistry and relationship to disease course. Clin. Cancer Res. 10, 8516–8520 10.1158/1078-0432.CCR-04-0885 15623633

[B151] Ying H. Elpek K. G. Vinjamoori A. Zimmerman S. M. Chu G. C. Yan H. (2011). PTEN is a major tumor suppressor in pancreatic ductal adenocarcinoma and regulates an NF-kappaB-cytokine network. Cancer Discov. 1, 158–169 10.1158/2159-8290.CD-11-0031 21984975 PMC3186945

